# Role of Translational Coupling in Robustness of Bacterial Chemotaxis Pathway

**DOI:** 10.1371/journal.pbio.1000171

**Published:** 2009-08-18

**Authors:** Linda Løvdok, Kajetan Bentele, Nikita Vladimirov, Anette Müller, Ferencz S. Pop, Dirk Lebiedz, Markus Kollmann, Victor Sourjik

**Affiliations:** 1 Zentrum für Molekulare Biologie der Universität Heidelberg, DKFZ-ZMBH Alliance, Heidelberg, Germany; 2 Institut für Theoretische Biologie, Humboldt Universität, Berlin, Germany; 3 Interdisziplinäres Zentrum für Wissenschaftliches Rechnen der Universität Heidelberg, Heidelberg, Germany; 4 Zentrum für Biosystemanalyze, Universität Freiburg, Freiburg, Germany; Johns Hopkins, United States of America

## Abstract

Chemotaxis allows bacteria to colonize their environment more efficiently and to find optimal growth conditions, and is consequently under strong evolutionary selection. Theoretical and experimental analyses of bacterial chemotaxis suggested that the pathway has been evolutionarily optimized to produce robust output under conditions of such physiological perturbations as stochastic intercellular variations in protein levels while at the same time minimizing complexity and cost of protein expression. Pathway topology in *Escherichia coli* apparently evolved to produce an invariant output under concerted variations in protein levels, consistent with experimentally observed transcriptional coupling of chemotaxis genes. Here, we show that the pathway robustness is further enhanced through the pairwise translational coupling of adjacent genes. Computer simulations predicted that the robustness of the pathway against the uncorrelated variations in protein levels can be enhanced by a selective pairwise coupling of individual chemotaxis genes on one mRNA, with the order of genes in *E. coli* ranking among the best in terms of noise compensation. Translational coupling between chemotaxis genes was experimentally confirmed, and coupled expression of these genes was shown to improve chemotaxis. Bioinformatics analysis further revealed that *E. coli* gene order corresponds to consensus in sequenced bacterial genomes, confirming evolutionary selection for noise reduction. Since polycistronic gene organization is common in bacteria, translational coupling between adjacent genes may provide a general mechanism to enhance robustness of their signaling and metabolic networks. Moreover, coupling between expression of neighboring genes is also present in eukaryotes, and similar principles of noise reduction might thus apply to all cellular networks.

## Abstract 2

Evolutionary selection for robustness of signaling output in the face of stochastic variations in protein expression may explain the organization of bacterial chemotaxis genes.

## Author Summary

All cellular networks are subject to fluctuations in the levels of their components. Robustness of the network output in the face of stochastic gene expression, or gene expression noise, is therefore essential to ensure proper function. Selection for robustness might thus have shaped much of the cellular evolution. We have used *Escherichia coli* chemotaxis, one of the most thoroughly studied model systems for signal transduction, to analyze the role of gene organization in robustness. Our mathematical modeling predicted that coupling the expression of chemotaxis proteins with opposing functions should buffer the output of the signaling pathway against stochastic variations in protein production. Consistent with this model, protein coexpression was indeed observed to improve chemotaxis and to be under selection during chemotaxis-driven spreading of a cell population. We show that tight coexpression is ensured by both transcriptional and translational gene coupling. We conclude that evolutionary selection for pathway robustness in the presence of gene expression noise can explain, not only the polycistronic organization of chemotaxis genes, but also the gene order within chemotaxis operons. Selection on the gene order was further confirmed by the observation of a strong bias towards specific pairwise occurrences of chemotaxis genes in sequenced prokaryotic genomes.

## Introduction

Any intracellular network is permanently exposed to a wide range of intra- and extracellular perturbations that affect levels of components and reaction rates. Both eukaryotic and prokaryotic systems have therefore evolved mechanisms that allow them to produce a robust output under varying conditions. In prokaryotes, the best-studied model system for signaling and robustness is the chemotaxis pathway of *E. coli* [1],[2]. The pathway includes transmembrane receptors (also called methyl-accepting chemotaxis proteins, or MCPs) of five types, the receptor-coupled kinase CheA, the adaptor CheW, the response regulator CheY, and the phosphatase CheZ, as well as the adaptation system that consists of two opposing receptor modification enzymes, the methyltransferase CheR and the methylesterase CheB. CheA autophosphorylation activity is controlled by ligand binding to receptors, with CheW needed to couple CheA to receptors. Phosphorylated CheA rapidly transfers the phosphate group to CheY, which controls direction of flagellar motor rotation and thereby bacterial swimming behavior. Phospho-CheY (CheY-P) dephosphorylation is accelerated by CheZ. Cells adapt to a constant stimulation by adjusting levels of receptor methylation, with higher methylated receptors being more efficient in kinase activation.

Robustness of the pathway output—the concentration of CheY-P—against varying levels of ambient stimulation and against intercellular variation in gene expression, or gene expression noise, is ensured by specific features of the pathway topology. Robust adaptation to a wide range of stimulus strength is achieved by an integral feedback from an activity state of receptors (kinase-activating vs. kinase-inactivating) to the methylation system, whereby CheR preferentially methylates inactive receptors and CheB demethylates active receptors [3]–[6]. On the other hand, robustness against natural intercellular variation in protein levels, or gene expression noise, primarily relies on the balance of opposing enzymatic activities, CheR/CheB and CheA/CheZ [7]. Such balance can perfectly compensate for the concerted expression noise, and it has been shown that the topology and reaction rates of the pathway are such that its output remains invariant under perfectly coupled overexpression of all chemotaxis proteins [7]. Robustness against expression noise is further improved by a negative phosphorylation feedback from the active CheA to CheB, which greatly enhances enzymatic activity of the latter, and partly compensates for both concerted and uncorrelated variations in protein expression.

These model predictions are consistent with the experimentally observed high correlation in the levels of individual chemotaxis proteins [7], which can be partly attributed to the gene organization in polycistronic transcriptional units, or operons, in which multiple genes are transcribed as one mRNA. Chemotaxis genes are organized into two operons: *mocha*, which encodes CheA and CheW along with flagellar motor proteins, and *meche*, which encodes two receptors—Tar and Tap—as well as CheR, CheB, CheY, and CheZ, whereas three other receptors are encoded elsewhere in the genome. However, even *cheA* and *cheY* genes that do not belong to the same operon show strong correlation in their single-cell expression levels, suggesting that a large part of gene expression noise originates at the upper level of transcriptional hierarchy that controls expression of all chemotaxis and flagellar genes [7].

Despite its success in accounting for robustness against concerted overexpression of all proteins, our previous computer model could not explain robustness against the experimentally observed degree of uncorrelated variation in protein levels in the population and predicted larger variation of the motor bias in the population than observed when identical levels of intercellular variation were assumed for all chemotaxis proteins [7]. This discrepancy indicated presence of additional robustness mechanisms, and in this work, we propose that translational coupling between adjacent genes on the *meche* and *mocha* operons represent such a mechanism. Translational coupling—defined as the interdependence of translation efficiency of neighboring genes encoded by the same polycistronic mRNA—has been previously described in *E. coli* [8]–[11], and can help to maintain a constant ratio between proteins expressed from the same operon. We experimentally demonstrated coupling for most pairs of chemotaxis genes in *E. coli* and confirmed that coexpression of these genes improves chemotactic performance. Computer simulations confirmed that negative effects of the uncorrelated expression noise can be reduced by genomic order of chemotaxis genes, in agreement with the gene arrangement in *E. coli*. Evolutionary importance of noise reduction mediated by translational coupling was further confirmed by strong bias towards particular pairwise coupling order of chemotaxis genes in bacterial genomes.

## Results

### Translational Coupling between Chemotaxis Genes

**Figure 1:**
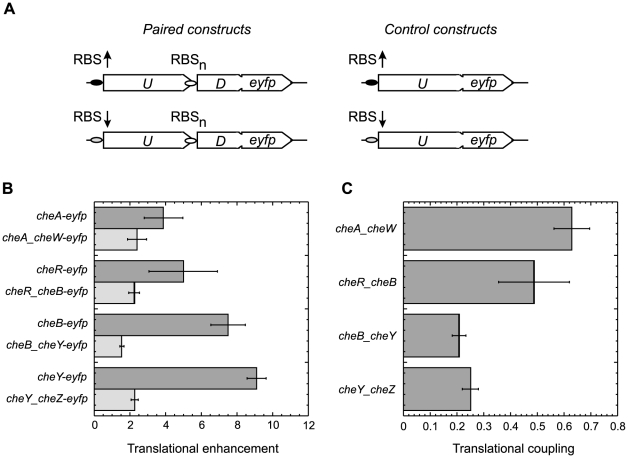
Translational coupling between neighboring genes. (A) Experimental strategy. Bicistronic constructs that contained pairs of neighboring chemotaxis genes in their chromosomal arrangement (*U*, upstream gene; *D*, downstream gene) were cloned under RBSs of different strength as indicated to create a C-terminal YFP fusion (*eyfp*, enhanced YFP gene) to a downstream gene. Strong RBS is indicated by a black oval and an up arrow, weak RBS by a grey oval and a down arrow. As a control of the RBS strength, the same sequence was placed in front of the monocistronic YFP fusion to the upstream gene. Downstream gene is under control of its native RBS (RBS_n_, open oval). Expression of the constructs was analyzed using FACS as described in Materials and Methods. (B) Direct (dark-grey) and indirect (light-grey) up-regulation of expression level of the fusion reporter by the stronger RBS, defined as the ratio of expression of constructs with the strong RBS to expression of corresponding constructs with the weak RBS. For the *cheA/cheW* pair, translation was regulated by using constructs that express either only short version of CheA or both long and short versions (see text for details). The values of up-regulation at varying (0 to 50 µM) levels of IPTG induction did not differ significantly and were averaged. (C) Translational coupling, defined as the ratio of indirect to direct up-regulation of expression levels by the stronger RBS. Error bars in (B and C) indicate standard deviations.

To test whether expression of neighboring chemotaxis genes might be coupled on a translational level, we analyzed three pairs of genes, *cheR_cheB*, *cheB_cheY*, and *cheY_cheZ*, from the *meche* operon, and one pair, *cheA_cheW*, from the *mocha* operon. Gene pairs were cloned as they appear in the genome, and the second gene was fused to a *eyfp* reporter (encoding yellow fluorescent protein, or YFP). The level of translation of the first gene was then selectively varied by placing ribosome-binding sites (RBSs) of different strength in front of it. As a control of the RBS strength, *eyfp* fusion to the first gene in the pair was placed under the same RBSs (Figure 1A). Thus determined differences in the RBS strengths varied from five to nine (Figure 1B) and were independent of the levels of IPTG-induced transcription (unpublished data). For the *cheA_cheW* pair, this strategy was complicated by the fact that CheA is expressed from two alternative translation initiation codons, yielding a long and a short version, CheA_L_ and CheA_S_, respectively [12]. Consequently, changing the strength of the first RBS had only a moderate effect on the total expression level of CheA. Instead, we compared constructs expressing CheA_L_ under the external RBS and CheA_S_ under the endogenous RBS with those expressing only CheA_S_ under the external RBS. The resulting net level of translation of CheA_L_-YFP and CheA_S_-YFP in the first construct was about four times higher than that of CheA_S_-YFP in the second construct.

For all pairs, stronger translation of the upstream gene resulted in an elevated expression of the downstream gene, implying the existence of a translational coupling (Figure 1B). The coupling was quantified as a ratio of the indirect up-regulation seen in constructs that carry gene pairs to the direct up-regulation of the first gene. The strength of translational coupling varied among gene pairs from approximately 0.2 to 0.6 (Figure 1C), apparently inversely correlating with the level of translational enhancement. Indeed, when an even stronger *cheR* RBS was used for the *cheR_cheB* pair to enhance translation approximately 30-fold, the observed coupling (∼0.2) was significantly weaker than the coupling at approximately 5-fold enhancement shown in Figure 1C. Such dependence may indicate saturation of coupling at high translational levels of the upstream gene, as expected if coupling results from the mRNA unfolding (see Discussion).

### Pairwise Coexpression of Genes Improves Chemotaxis

**Figure 2:**
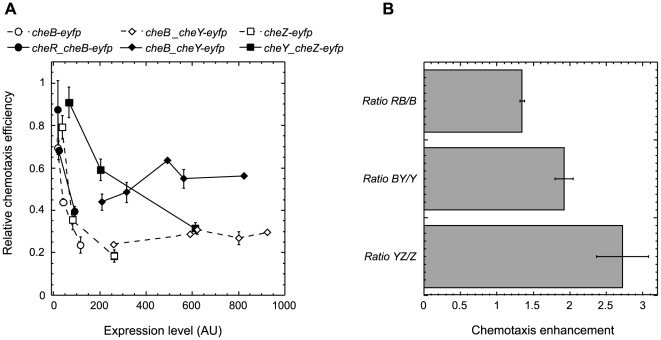
Improvement of chemotaxis by coexpression of signaling proteins. (A) Dependence of the chemotaxis-driven spreading of bacteria on soft agar (swarm) plates on the protein expression level for monocistronic (open symbols, dashed lines) or bicistronic (filled symbols, solid lines) constructs. Protein expression from pTrc99A-based plasmids pVS138 (*cheB-eyfp*) and pVS145 (*cheR*_*cheB-eyfp*) in strain RP4972 (*ΔcheB*) and pVS64 (*cheZ-eyfp*) and pVS305 (*cheY_cheZ-eyfp*) in strain VS161 (*ΔcheZ*) was induced by 10, 25, or 100 µM IPTG. A nontranslated 316-nucleotide fragment of *cheB* was included upstream of the *cheY* start codon in pLL33 (−316_*cheY-eyfp*) plasmid to achieve expression comparable to pLL36 (*cheB*_*cheY-eyfp*) construct (see Materials and Methods for details), and both constructs were expressed in strain VS100 (*ΔcheY*) under weaker pBAD promoter induced by 0%, 0.0005%, 0.001%, 0.003%, 0.005%, or 0.01% arabinose. Expression levels were measured in liquid cultures grown under the same induction as described in Materials and Methods. Chemotaxis efficiency was determined as the size of a swarm rings and normalized to that of wild-type strain RP437 transformed with either a pTrc99A (for pVS138, pVS145, pVS64, and pVS305) or a pBAD33 (for pLL33 and pLL36) vector. (B) Enhancement of chemotactic efficiency by expression coupling. Enhancement was calculated as a ratio of chemotaxis efficiency at a given expression level of the monocistronic construct to the interpolated efficiency at the same expression level of the YFP fusion in the respective bicistronic construct in (A), and values at different expression levels were averaged. Error bars indicate standard deviations.

Maintaining a constant ratio between signaling proteins may be important for a proper functioning of the chemotaxis pathway under varying protein levels, and we have recently shown that the chemotaxis system is much less sensitive to a concerted overexpression of CheY and CheZ than to the overexpression of each of these proteins individually [13]. We thus tested whether a coexpression of the proteins from bicistronic constructs will improve performance of the pathway in a chemotaxis-driven spreading of bacteria in soft agar (Figure 2). Indeed, cells that express a YFP fusion to a particular gene as a monocistronic construct in the respective knock-out strain spread less efficiently than the cells that express this fusion as a downstream gene in bicistronic constructs at the same level (Figure 2A), with a clear enhancement of chemotaxis that resulted from gene coexpression (Figure 2B).

**Figure 3:**
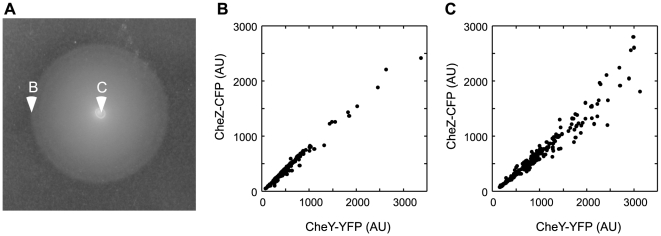
Chemotactic selection for posttranscriptional coupling. (A) Chemotaxis-driven spreading of VS104 [*Δ(cheYcheZ)*] cells expressing CheY-YFP and CheZ-CFP from a bicistronic construct pVS88 on soft agar (swarm) plates. (B and C) Scatter plots of single-cell levels of CheY-YFP and CheZ-CFP in cells taken from the edge (B) and from the middle (C) of the spreading colony. Relative concentrations of fluorescent proteins in individual cells were determined using fluorescence microscopy as described in Materials and Methods. Protein expression was induced with 17 µM IPTG; data for 10 µM IPTG induction are shown as supporting Figure S3. AU, arbitrary units.

Such enhancement suggests that the coexpression of particular chemotaxis genes should be evolutionary selected, although it does not specifically distinguish between translational and transcriptional coupling. To directly test whether there is a chemotaxis-driven selection for the expression coupling beyond cotranscription, we compared single-cell levels of CheY-YFP and CheZ fused to cyan fluorescent protein, CheZ-CFP, that were expressed from one bicistronic construct in *E. coli* population spreading in soft agar (Figure 3 and Figure S3). Best-chemotactic cells at the front edge of the spreading ring (Figure 3A and Figure S3A) showed very strong correlation between the levels of both proteins (Figure 3B and Figure S3B). In contrast, the correlation in cells that remained behind and were not selected for chemotaxis was significantly weaker (Figure 3C and Figure S3C), despite the fact that both subpopulations express CheY-YFP and CheZ-CFP from the same bicistronic mRNA. This demonstrates chemotactic selection for the posttranscriptional coupling between protein levels and supports our assumption that translational coupling should be evolutionary beneficial.

### Translational Coupling between Selected Genes Is Predicted to Enhance Robustness of the Pathway

**Figure 4:**
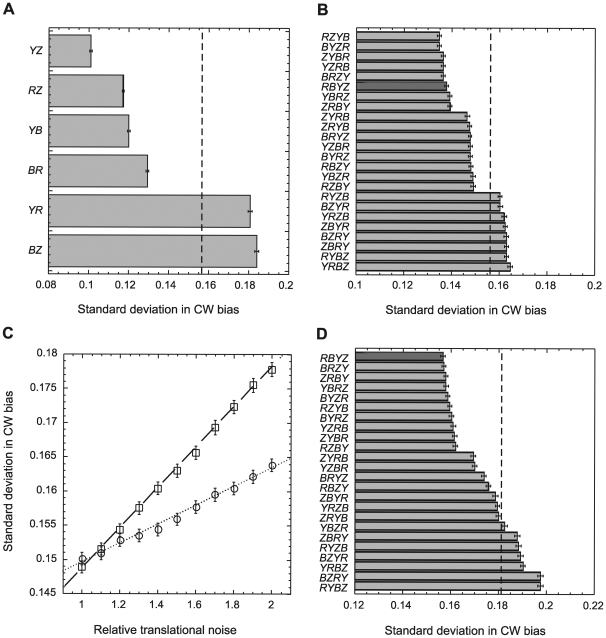
Simulated effects of translational coupling on robustness of the signaling output. Standard deviation of the CW motor bias in a population of 10^5^ cells was simulated in presence of gene expression noise as described in Materials and Methods and in supporting information (Text S1). (A) Simulations for 100% pairwise coupling of indicated chemotaxis genes, with remaining genes being uncoupled. (B) Simulations for different arrangements of translationally coupled chemotaxis genes, performed at equal noise levels for all genes and 25% coupling. (C) Asymmetric effects of translational noise for 25% coupling between *cheR*_*cheZ* (circles, dotted line) and *cheZ*_*cheR* (squares, dashed line). Linear fits to the data are guide to the eye. (D) Simulations for different gene orders as in (B), at 1.5-fold higher noise for the weakly expressed *cheR* and *cheB* genes. Dark-grey bars indicate gene order in *E. coli*. Standard deviation of CW bias in absence of coupling is indicated by vertical dashed lines. Genes are indicated by single letters, i.e., Y = CheY, and so forth. Error bars indicate confidence intervals.

Why are some proteins and not the others coupled through sequential gene arrangement in one operon? As mentioned above, enhanced robustness against uncorrelated gene expression noise—resulting from stochasticity of translation—is the most likely mechanism by which translational coupling could benefit chemotaxis. We thus used computer simulations to test whether preferential pairing of particular chemotaxis genes and the resulting gene order on the chromosome can improve robustness of the pathway output—adapted clockwise (CW) rotation bias of flagellar motor—against translational noise when translational coupling is taken into account. Considering four genes *cheR*, *cheB*, *cheY*, and *cheZ*, our in silico chemotaxis network model indeed confirmed that positive correlations between expression of adjacent genes via translational coupling affect deviations from the optimal adapted CW bias within a population (Figure 4). Simulating a 100% pairwise translational coupling between particular genes in the background of uncorrelated fluctuations of all other genes (Figure 4A) showed favorable reduction in the standard deviation of CW bias for four adjacent gene pairs—*cheY_cheZ*, *cheR_cheZ*, *cheY_cheB*, and *cheR_cheB*. Note that because of the perfect coupling, the gene order in these simulations is not important, so that *cheY_cheZ* and *cheZ_cheY* pairs are equivalent. In all these cases, a positive effect is observed whenever a gene that enhances CheY-P level upon overexpression is coupled to a gene that reduces CheY-P level upon overexpression or vice versa (see Discussion). A negative effect—the increased variation in CW bias—was observed by coupling *cheY_cheR* and *cheB_cheZ* genes that have similar effects on the CheY-P level.

We next investigated which overall order of chemotaxis genes would yield the optimal noise reduction based on the observed preferences in pairwise gene coupling. When levels of translational noise and coupling efficiency were assumed to be equal for all four genes, 16 gene orders out of possible 24 permutations were predicted to reduce variation of the bias in the population compared to the simulation in absence of coupling, whereas eight gene orders increased that variation (Figure 4B). The degree of noise reduction or enhancement in this case was largely the consequence of maximizing favorable pairings and minimizing unfavorable pairings. Eight gene orders with three positive couplings—including the native gene order in *E. coli*—showed the most pronounced noise reduction. Additional weak gradation in the ranking resulted from the pair-specific differences in the extent of noise reduction or enhancement (Figure 4A), with the *cheY_cheZ* (or *cheZ_cheY*) pair being present in all of the highest ranked orders. The detailed ranking among arrangements with the same number of positive couplings depended only weakly on the reaction rates in the pathway but strongly on the strength of translational noise. For different gene-specific levels of translational noise, the optimal gene order becomes dependent not only on the number of positive pairs but also on their sequence, due to asymmetric effects of coupling on the output noise (Figure 4C; see Text S1 for details). As a result, in a more physiological case of 1.5-fold higher noise in expression of the weakly translated genes CheR and CheB (Figure 4D) the ranking of gene orders becomes more differentiated, with the native order of chemotaxis genes in *E. coli* providing the largest noise reduction.

### Consensus Order of Chemotaxis Genes in Bacteria

**Figure 5:**
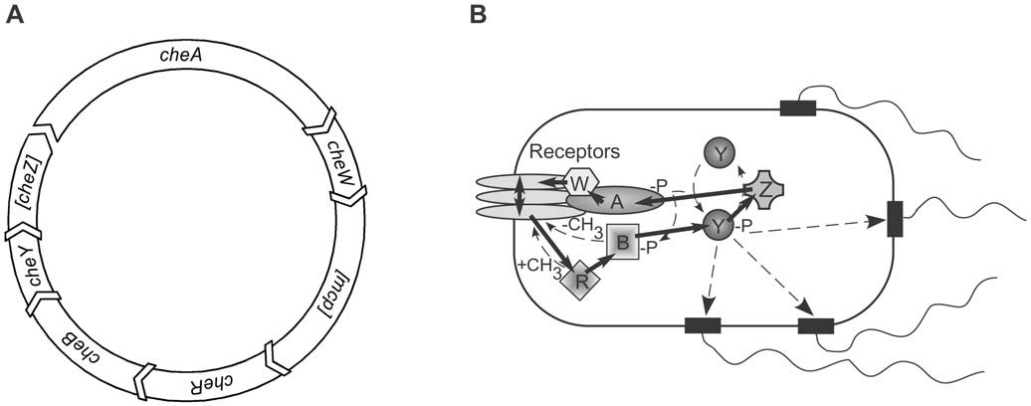
Genetic coupling of chemotaxis genes in bacteria. (A) Preferential order of pairwise chemotaxis gene coupling among analyzed bacteria. Receptor (*mcp*) gene is shown in brackets because the number of receptor genes between *cheW* and *cheR* is variable; *cheZ* is shown in brackets because it is only present in a subset of bacteria. See Table 1 and Tables S2 and S3 for the frequencies of relative occurrence. (B) Genetic coupling (solid arrows) among chemotaxis proteins shown for *E. coli* pathway. Thin dashed arrows denote pathway reactions and CheY-P binding to flagellar motor.

Our analyses imply that the order of chemotaxis genes coupling on the chromosome should be subject to evolutionary selection and therefore conserved among bacteria. A comprehensive analysis of 824 sequenced bacterial genomes, 527 of which contain annotated chemotaxis genes (Table S1, Text S2), confirmed existence of a strong bias in the pairwise co-occurrence of these genes in the genome and in their order (Table 1). The resulting consensus order (Figure 5A) was consistent with the modeling predictions and showed a nearly perfect match to the chemotaxis gene arrangement in *E. coli*. Because our mathematical model explicitly includes the phosphatase CheZ, which is only found in a subset of 200 bacterial species, gene coupling in genomes with and without *cheZ* was also analyzed separately (Tables S2 and S3, respectively). Both yielded essentially the same consensus gene order, except for weaker coupling between *cheB* and *cheY* in absence of *cheZ*. This confirms that selection for other pairs does not depend on specific mechanism of CheY dephosphorylation. Notably, the overall gene order in individual prokaryotes, including those with most studied chemotaxis systems [14], is only conserved among closely related species (Figure S1). This suggests—in agreement with the results of our modeling analysis—that it is primarily the pairwise gene coupling rather than the consensus as a whole that is under selection.

**Table 1:** Absolute frequencies^a^ of a pairwise occurrence of chemotaxis genes in 527 genomes containing at least one chemotaxis gene.

Gene	*cheA* (771)		*cheW* (1,232)		*cheR* (802)		*cheB* (656)		*cheY* (1,376)		*cheZ* (209)		*mcp* b (6,521)	
	left	right	left	right	left	right	left	right	left	right	left	right	left	right
*cheA*	1.0	<1	**19.6**	3.2	2.7	2.2	14.8	8.6	<1	7.7	<1	**32.5**	<1	<1
*cheW*	7.4	**37.8**	5.9	5.6	**20.8**	7.2	5.2	1.4	2.3	2.8	0.0	0.0	4.0	3.0
*cheR*	2.3	3.9	4.6	13.7	<1	<1	**28.6**	10.7	1.9	<1	0.0	0.0	<1	2.0
*cheB*	5.2	15.1	<1	2.7	8.6	**26.1**	<1	<1	**7.2**	2.3	<1	0.0	<1	<1
*cheY*	**15.7**	<1	3.4	2.3	1.4	3.1	4.9	**15.0**	1.9	1.7	**90.0**	0.0	<1	<1
*cheZ*	8.1	<1	0.0	0.0	0.0	0.0	0.0	0.0	<1	**9.6**	0.0	0.0	<1	0.0
*mcp*	10.5	6.4	13.0	**16.5**	16.8	2.1	1.1	2.3	1.9	1.2	0.0	<1	**5.3**	**5.1**

a Absolute frequencies were calculated as the number of gene occurrences in −1 (left neighbor) or +1 (right neighbor) positions relative to a reference gene, normalized by the total number of reference gene counts (shown in parentheses). Strongest genomic coupling on each side (highest co-occurrence frequency) is marked in bold.

b Genes encoding chemoreceptors (methyl-accepting chemotaxis proteins).

Additional statistical analysis of distances between neighboring chemotaxis genes (Figure S2) confirmed that most frequently coupled genes are typically close enough to each other, less than 30 nucleotides, to allow a simultaneous ribosome interaction with the stop codon of upstream gene and the RBS of the downstream gene, and are thus likely to be translationally coupled. The only exceptions are *mcp*_*mcp* and *cheW*_*mcp* pairs that are frequently separated by a larger intergenic distance. Such separation is consistent with genetic organization in *E. coli*, where *cheW* and the downstream *mcp* (*tar*) belong to different operons, and three receptor genes are uncoupled from the chemotaxis operons.

## Discussion

### Translational Coupling as a Mechanism of Noise Reduction

Intercellular variation in protein levels in a genetically homogeneous cell population, or gene expression noise, is the major source of perturbations that affect performance of all cellular pathways. In prokaryotes, as in eukaryotes, the largest part of this noise appears to originate from fluctuations of global factors that affect expression of all genes in a cell, and from stochastic variations in promoter activity [15]–[18]. Since bacterial genes of related function are typically transcriptionally coupled through the polycistronic gene organization and common regulation, concerted variations in the levels of related genes are therefore expected to be the dominant type of the expression noise. Strong correlation in the single-cell levels of individual chemotaxis proteins has been indeed observed in *E. coli*, and the chemotaxis pathway was shown to be primarily robust against such concerted variation [7].

However, stochasticity of translation results in significant uncorrelated variation in the levels of two proteins produced from one polycistronic mRNA [7], and it is thus not surprising that bacteria evolved mechanisms to reduce effects of such translational noise. Translational coupling between bacterial genes in operons has been described before, primarily in metabolic operons [10],[11],[19]–[21], but also between genes encoding ribosomal proteins [8] and a two-component sensor [9]. Such coupling mostly happens when the stop codon of the upstream gene is close to or overlaps with the start codon or with the Shine-Dalgarno (SD) sequence of the downstream gene. Translational coupling may result from a combination of several factors. First, translation of the upstream gene will locally increase the number of ribosomes close to the initiation codon of the downstream gene, which could then efficiently reinitiate translation of the downstream gene even in absence of a strong SD sequence [20]. Second, ribosomes translating the upstream gene will also unwind any secondary structure of the mRNA that might form around the SD sequence of the downstream gene, as long as this sequence belongs to the translated region of the upstream gene. Such opening of the SD sequence will facilitate both reinitiation of translation by already bound ribosomes and entry of new ribosomes [19]. The latter mechanism is supported by the observed inverse correlation of coupling with the translation strength, since in this case, coupling is expected to saturate as soon as the mRNA is completely unfolded. Whatever the mechanism of coupling is, it has been proposed to enable a tighter control of the stoichiometry of protein complexes [10].

### Selection for Robustness Can Explain Order of Chemotaxis Genes

Our experimental results and computational analyses suggest that—along with the robust pathway topology and transcriptional coupling between chemotaxis genes—translational coupling is yet another factor that contributes to the robustness of signaling in chemotaxis. Functional importance of the tight pairwise coupling between protein levels was demonstrated by the improvement of chemotaxis when any of tested endogenous pairs was expressed from one bicistronic construct. Furthermore, selection for the enhanced posttranscriptional coupling between protein levels was observed in cells that were spreading most efficiently in a chemotaxis assay. Translational coupling appears to specifically compensate the output level of CheY-P and thereby CW motor bias against stochastic variations in translation of individual genes. In silico analysis demonstrated higher robustness of particular arrangements of chemotaxis genes against translational noise, namely those that maximize the number of gene couples with opposing effects on the CheY-P level. Although better knowledge of modeling parameters would be required to definitively resolve relative positions of the gene orders with highest ranking within our model, *E. coli* gene order ranked best for output robustness when we assumed that the weakly translated genes *cheR* and *cheB* have slightly higher (1.5-fold) noise levels than the more efficiently translated genes *cheY* and *cheZ*. Thus, both modeling and experiments suggest that *E. coli* gene order is likely to have evolved under pressure to maximize coupling between expression of antagonistic proteins, and thereby robustness of the pathway output. This idea is further supported by the observation that the order of chemotaxis genes in bacterial genomes is not random, with a strong bias towards the same gene coupling as in *E. coli*.

Selection for coupling in all studied *E. coli* gene pairs can be explained based on the known properties of the chemotaxis pathway (Figure 5B). CheA and CheW form a stable complex with chemotaxis receptors [22],[23]. The stoichiometry and functional properties of this complex are affected by the relative levels of individual proteins [24],[25], and relative translation of CheA and CheW is thus expected to be under a tight control. Coupling between expression of CheY and CheZ serves to reduce the level of CheY-P when CheY is up-regulated, by increasing the level of phosphatase and thereby returning the pathway to homeostasis. Inversely, coupling could increase the rate of CheY phosphorylation when CheZ is up-regulated. Coupling between the levels of CheR and CheB is also expected to increase robustness of the CheY-P output, since these proteins form a pair of counteracting enzymes that control the steady-state level of receptor methylation and, as a consequence, that of kinase activity. From the point of robustness, coupling between CheB and CheY is not surprising either. On one hand, these two proteins compete for CheA-dependent phosphorylation, including stimulation-dependent competitive binding at the P2 domain of CheA [26],[27]. On the other hand, higher CheB activity reduces the level of receptor methylation and thereby the rate of CheY phosphorylation. A coelevated level of CheY would thus counteract an increase in the level of CheB both directly, by reducing CheB phosphorylation, and indirectly, by increasing the level of phospho-CheY. Similarly, the up-regulation of CheB should counterbalance an increased level of CheY.

In addition to these pairs, our bioinformatics analysis revealed a strong coupling between receptor (*mcp*) genes and *cheW*, in agreement with these gene products being parts of the same stable signaling complex. This coupling is stronger than that between receptors and *cheA*, apparently consistent with a role of CheW as an adapter between receptors and CheA [22]. Coupling between *cheZ* and *cheA*, which is also statistically significant in *cheZ*-containing genomes, could serve a similar function as the coupling between *cheY* and *cheZ*, and compensate for an increase in the level of phosphatase by an increase in the kinase activity. A compensatory effect on noise is also expected for the coupling between *cheA* and *cheB*, since CheB provides a negative feedback to the kinase activity. The reason for coupling between receptor genes (or *cheW*) and *cheR* is less obvious, but keeping a proper ratio between receptors and methyltransferase activity might be important for maintaining a constant steady-state level of receptor methylation. Significant coupling between *cheY* and *cheA* resembles translation coupling observed in other two-component systems, although theoretical analysis suggests that such coupling should only take place when—like in these other systems—the kinase is bifunctional, i.e., has a phosphatase activity [28]. This prediction remains to be experimentally tested for bacterial chemotaxis systems.

### Evolution of Gene Order in Chemotaxis Operons

In agreement with our mathematical model, pairwise coupling between particular chemotaxis genes rather than the gene order as a whole appears to be primarily under evolutionary selection, with the overall gene order being conserved only among closely related species. It is thus unlikely that the observed consensus is a consequence of the conservation—or lateral transfer—of the same chemotaxis operon across prokaryotes. Individual genes appear to have been rearranged multiple times throughout the evolution, with differences in gene order between groups of closely related species possibly reflecting variations in the pathway topology and gene regulation.

Proposed robustness-driven mechanism of gene ordering in operons can be seen as a refinement of the models that explain operon formation by positive selection for the coregulation of genes encoding components of the same pathway or of one multicomplex [29]. Particularly, it is closely related to the previously discussed balance hypothesis [30],[31], which postulates that an imbalance in the concentrations of two subcomponents of a multiprotein complex can result in the formation of nonfunctional complexes with wrong stoichiometry and will be therefore under negative evolutionary selection. The balance hypothesis can be well used, for example, to explain the polycistronic organization of metabolic genes, which indeed frequently encode components of multisubunit enzymes. In case of chemotaxis, strong coupling between *cheA* and *cheW* presumably results from similar constrains. However, our model does not require that proteins form stable complexes, or even directly interact with each other, to have mutually compensatory effects on the output and thus to benefit from coupling. At the same time, we predict that coupling of other proteins in the pathway can be detrimental and thus under negative selection. Our analysis thus extends the regulation-based model of operon formation to explain the internal operon structure.

Although our model does not describe the process of chemotaxis operon formation itself, evolutionary selection for the gradual increase in proximity of chemotaxis genes through genome rearrangements seems to be the most likely mechanism. Due to the correlation in expression of bacterial genes that are close on the chromosome [32],[33], such increase in proximity would lead to the gradual increase in gene coupling and thereby in robustness of the pathway output. Additional selection for the lateral gene cotransfer, as proposed by the selfish operon model [34], might be also involved in the initial grouping of chemotaxis genes. However, because in this case transferred genes as a group must provide an immediate benefit to the host, selfish operon model would require grouping and cotransfer of multiple genes involved in flagellar assembly and would therefore not explain emergence of selective pairing between chemotaxis genes.

### Conclusions

Taken together, our results emphasize the importance of translational coupling and gene order in the overall organization of the chemotaxis pathway in *E. coli* and other bacteria. Strong bias towards a particular order of genes on the chromosome was predicted by our computer simulations assuming selection for robustness of the pathway output against gene expression noise, and confirmed by the bioinformatics' analysis of sequenced bacterial genomes. Such organization is evolutionary beneficial because it improves robustness of the signaling output without adding a cost of the increased complexity and is thus expected to be ubiquitous in bacterial networks. Although translational coupling is absent in eukaryotes, expression levels of neighboring genes are frequently coupled on the level of chromatin remodeling [35],[36]. Moreover, it has been recently proposed that segregation of eukaryotic genes into particular chromosomal regions is driven by the reduction in gene expression noise [37]. The gene order on the chromosome may thereby contribute to network robustness in all organisms.

## Materials and Methods

### Strains and Plasmids

*E. coli* K-12 strains used in this study were derived from RP437 [38]. All strains and plasmids are summarized in Tables 2 and 3. Monocistronic constructs expressing YFP fusions to CheR, CheB, CheY, CheZ, and CheA under moderately strong RBSs and pTrc promoter inducible by isopropyl β-d-thiogalactoside (IPTG) have been described before [13],[26],[39]–[41]. They were used to obtain constructs with strong RBSs (summarized in Table 4) and bicistronic constructs by using PCR and cloning to modify the upstream sequence. Because expression of *cheY* is strongly up-regulated by a sequence inside *cheB* gene (A. Müller and V. Sourjik, unpublished data), a nontranslated 316-nucleotide fragment of *cheB* was included upstream of the *cheY* start codon in pVS319 (−316_*cheY-eyfp*) plasmid to achieve expression comparable to pVS142 (*cheB*_*cheY-eyfp*) construct. To reduce levels of expression for the *cheB*_*cheY-eyfp* and −316_*cheY-eyfp* constructs, both fragments were cloned under weaker pBAD promoter inducible by l-arabinose, to obtain pLL33 and pLL36, respectively.

**Table 2:** Strains used in this study.

Strain	Description/Relevant Genotype	Reference
RP437	Wild type for chemotaxis	[38]
VS100	*ΔcheY*	[40]
VS104	*Δ(cheYcheZ)*	[41]
VS161	*ΔcheZ*	[13]
RP4972	*ΔcheB*	J. S. Parkinson, personal gift

**Table 3:** Plasmids used in this study.

Plasmid	Description^a^	Reference
pTrc99A	Expression vector; pBR ori, pTrc promotor, Amp^R^	[45]
pBAD33	Expression vector; pACYC ori, pBAD promotor, Cm^R^	[46]
pDK57	RBS^CheYS2^_CheA_S_-YFP expression plasmid; pTrc99a derivate	[26]
pDK66	Expression vector for cloning of C-terminal YFP fusions; RBS^CheYS^ pTrc99a derivative	[47]
pVS18	RBS^CheY^_CheY-YFP expression plasmid; pTrc99a derivate	[41]
pVS64	RBS^CheZ^_CheZ-YFP expression plasmid; pTrc99a derivate	[39]
pVS88	RBS^CheY^_CheY-YFP_ RBS^CheZ^_CheZ-YFP bicistronic construct; pTrc99a derivate	[25]
pVS137	RBS^CheR^_CheR-YFP expression plasmid; pTrc99a derivate	[13]
pVS138	RBS^CheB^_CheB-YFP expression plasmid; pTrc99a derivate	[13]
pVS142	RBS^CheB^_CheB_CheY-YFP expression plasmid; pTrc99a derivate	This work
pVS145	RBS^CheR^_CheR_CheB-YFP expression plasmid; pTrc99a derivate	This work
pVS261	RBS^CheYS^_CheA-YFP expression plasmid; pTrc99a derivate	This work
pVS305	RBS^CheY^_CheY_CheZ-YFP expression plasmid; pTrc99a derivate	This work
pVS319	−316_CheY-YFP expression plasmid; pTrc99a derivate	This work
pVS321	RBS^CheY↑^_CheY_CheZ-YFP expression plasmid; pTrc99a derivate	This work
pVS450	RBS^CheB↑^_CheB_CheY-YFP expression plasmid; pTrc99a derivate	This work
pVS451	RBS^CheR↑↑^_CheR_CheB-YFP expression plasmid; pTrc99a derivate	This work
pVS452	RBS^CheR↑↑^_CheR-YFP expression plasmid; pTrc99a derivate	This work
pVS487	RBS^CheB↑^_CheB-YFP expression plasmid; pTrc99a derivate	This work
pVS490	RBS^CheYS2^_CheA_CheW-YFP expression plasmid; pTrc99a derivate	This work
pVS495	RBS^CheY↑^_CheY-YFP expression plasmid; pTrc99a derivate	This work
pVS520	RBS^CheYS^_CheA_S__CheW-YFP expression plasmid; pTrc99a derivate	This work
pAM80	RBS^CheR↑^_CheR-YFP expression plasmid; pTrc99a derivate	This work
pAM81	RBS^CheR↑^_CheR_CheB-YFP expression plasmid; pTrc99a derivate	This work
pLL33	−316_CheY-YFP expression plasmid; pBAD33 derivate	This work
pLL36	RBS^CheB^_CheB_CheY-YFP expression plasmid; pBAD33 derivate	This work

a See Table 4 for description and exact sequence of RBS.

**Table 4:** Upstream ribosome binding sequences of the fusion constructs.

Construct	Upstream Sequence^a^
RBS^CheR^	*GAGCTC*TTGAGAAGGCGCT**ATG**
RBS^CheB^	*GAGCTC*AGTAAGGATTAACG**ATG**
RBS^CheY^	*GAGCTC*CGTATTTAAATCAGGAGTGTGAA**ATG**
RBS^CheZ^	*GAGCTC*CAGGGCATGTGAGGATGCGACT**ATG**
RBS^CheYS^	*ACTAGT*GAAGGAGTGTGCC**ATG**
RBS^CheR↑^	*GAGCTC*GATAGGGTGGGCGCT**ATG**
RBS^CheR↑↑^	*GAGCTC*GATAGGAAAGGCGCT**ATG**
RBS^CheB↑^	*GAGCTC*AAGAGGAAATTAACG**ATG**
RBS^CheY↑^	*GAGCTC*AATAGAGGAAATGTGAA**ATG**

A single upward arrow (↑) indicates an enhanced RBS; double arrows (↑↑) indicate a strongly enhanced RBS.

a Italic type indicates recognition site of restriction enzymes, SacI or SpeI, used for cloning the constructs; boldface font indicates the start codon.

### Growth Conditions

Overnight cultures were grown in tryptone broth (TB; 1% tryptone, 0.5% NaCl) containing ampicillin (100 µg/ml) or chloramphenicol (100 µg/ml) at 30°C for 16 h. For measurements of the YFP expression in liquid cultures, overnight cultures were diluted 1∶100 in fresh TB containing ampicillin and indicated concentrations of IPTG or l-arabinose. Cell cultures were allowed to grow 3.5–4 hours at 34°C in a rotary shaker until an optical density at 600 nm (OD_600_) of 0.45, then harvested by centrifugation (8,000 rpm, 1 min), washed, and then resuspended in tethering buffer (10 mM potassium phosphate, 0.1 mM EDTA, 1 µM l-methionine, 10 mM sodium lactate [pH 7]).

TB soft agar (swarm) plates were prepared by supplementing TB with 0.3% agar (Applichem), required antibiotics (100 µg/ml ampicillin; 34 µg/ml chloramphenicol), and indicated concentrations of IPTG and l-arabinose. Plates were inoculated using fresh cells from LB agar plates, and swarm assays were performed for 6–8 h at 34°C. Images of swarm plates were taken using a Canon EOS 300 D (DS6041) camera, and analyzed with ImageJ (Wayne Rasband, NIH) to determine the diameter of the swarm rings.

### Quantification of Gene Expression

Mean expression levels of fluorescent proteins were quantified in a population of approximately 10^4^ cells as described before [7] using flow cytometry on a FACScan (BD Biosciences) equipped with an argon 488-nm laser. FACScan data were analyzed using CellQuestTM Pro 4.0.1 software. Mean value of the autofluorescence background, measured for control cells, was subtracted from all values. Single-cell protein levels were measured using fluorescence microscopy on a Zeiss AxioImager Z1 microscope equipped with an ORCA AG CCD Camera (Hamamatsu) and HE YFP (Excitation BP 500/25; Dichroic LP 515; Emission BP 535/30) and HE CFP (Excitation BP 436/25; Dichroic LP 455; Emission BP 480/40) filter sets. Integral levels of fluorescence in individual cells were quantified using an automated custom-written ImageJ plug-in [13] and normalized to cell length to obtain relative concentrations of fluorescent proteins [42].

### Analysis of Gene Order

Analysis of the order of chemotaxis genes was performed using a custom-written Perl program. The program scanned text files of 824 microbial genomes from the GenBank database using variable regular expressions to identify chemotaxis genes in the annotation. Features which may contain information about the gene function (\gene, \function, \product, \note) were successively retrieved for every coding sequence (CDS) in a genome, recorded, and then analyzed for occurrence of chemotaxis terms. Because the description of chemotaxis genes was often periphrastic, we performed a preliminary manual analysis of selected genomes to determine the most frequently used and misused synonyms, which were further used to define positive and negative terms for automatic chemotaxis genes recognition. A chemotaxis gene was recognized if its annotation contained one of the positive terms that point to its specific function and did not contain negative terms which indicate that the gene function is ambiguous or related to another chemotaxis gene (Table S1). Identified genes were then verified manually by looking through their extracted annotations, to remove possible false-positive entries; this verification confirmed high efficiency of the annotation-based gene recognition. Only genes with clearly defined chemotaxis-related annotations were included in the final analysis. Additionally, we restricted our analysis to chemotaxis genes that are present in *E. coli*, which are well annotated and —with the sole exception of *cheZ*—conserved in most prokaryotes. Homologs of these genes were found in 527 genomes. Starting and ending nucleotide positions of each recognized chemotaxis gene as well as the upstream and downstream neighboring genes were recorded. Names and genomic positions of all recognized chemotaxis genes are provided as supporting information (Text S2). The resulting gene duplets were analyzed to calculate co-occurrences of neighbors (Table 1 and Tables S2 and S3) and to determine intergenic distances (Figure S2).

Phylogenetic analysis of chemotaxis gene order in selected genomes (Figure S1) was performed using the Web-based program Composition Vector Tree (CVTree, http://cvtree.cbi.pku.edu.cn/), which constructs *phylogenetic trees* based on the organism's complete genomic sequence [43]. The resulting phylogenetic trees were plotted using a Java-based program Archaeopteryx (http://www.phylosoft.org/archaeopteryx/).

### Computer Simulations

To calculate the adapted level of free phosphorylated CheY, we simulated the pathway using differential equations based on mass action kinetics. Rates and binding constants are taken from in vitro and in vivo experiments (http://www.pdn.cam.ac.uk/comp-cell). The mathematical model includes all known protein interactions among CheR, CheB, CheY, and CheZ. The adapted receptor activity is determined by the methylation level and consequently by the ratio between receptor-bound CheR and CheB, allowing us to omit all details of transient adaptation kinetics. The relation of phosphorylated CheY to the flagellar motor rotation bias follows from the experimentally determined motor response curve [44]. Our mathematical model reflects the experimentally observed robustness of the pathway output against concerted overexpression of all chemotaxis proteins but shows the expected sensitivity to independent variations in protein levels. Effects of translational noise on protein concentration has been simulated by Gaussian random variables with means given by the measured wild-type concentrations and a common standard deviation over mean of 0.05 to arrive at the experimentally observed cell-to-cell variations of the CW bias [7]. The strength of translational coupling constant was set to 25% of the mean translational efficiency to generate the rank list (Figure 4). The error bars in Figure 4 indicate the 95% confidence intervals for the standard deviation of the CW bias for a cell population of 10^5^ individuals, resulting from data resampling using bootstrap. The influence of transcriptional noise or extrinsic noise on the gene order was not significant as both CheY-P level of our chemotaxis pathway model and experimentally measured CW rotation bias [7] are almost insensitive to increased transcriptional activity. The details of mathematical model are provided as supporting information (Text S1)

## Supporting Information

Figure S1 **Phylogenetic map of chemotaxis gene order in selected prokaryotes.** Order of chemotaxis genes in selected prokaryotes was mapped on the phylogenetic tree, constructed as described in Materials and Methods. Receptor genes or *mcp* are indicated by m, *cheA* by A, *cheB* by B, and so on. A minus sign (−) indicates hypothetical protein of unknown function or protein unrelated to chemotaxis. Independent gene groups are separated by dots.
(0.44 MB PDF)

Figure S2 **Pairwise distances between the most frequently neighboring chemotaxis genes over 527 genomes.** Distance between neighboring chemotaxis genes was defined as the number of nucleotides between the last nucleotide of the stop codon of the upstream gene and the first nucleotide of start codon of the downstream gene. Intergenic distances were determined as described in Materials and Methods, and plotted as histograms.
(0.47 MB PDF)

Figure S3 **Chemotactic selection for posttranscriptional coupling of CheY-YFP and CheZ-CFP at 10 µM IPTG induction.** (A) Chemotaxis-driven spreading of VS104 [*Δ(cheYcheZ)*]/pVS88 cells on soft agar (swarm) plates. (B and C) Scatter plots of single-cell levels of CheY-YFP and CheZ-CFP in cells taken from the edge (B) and from the middle (C) of the spreading colony. Relative concentrations of fluorescent proteins in individual cells were determined using fluorescence microscopy as described in Materials and Methods. See description of Figure 3 in the main text for more details.
(0.62 MB PDF)

Table S1 **Terms used for identification of chemotaxis genes.**
(0.07 MB DOC)

Table S2 **Pairwise occurrence of chemotaxis genes in 200 genomes containing** ***cheZ*** **.**
(0.05 MB DOC)

Table S3 **Pairwise occurrence of chemotaxis genes in 327 genomes without** ***cheZ*** **.**
(0.05 MB DOC)

Text S1 **Mathematical model.**
(0.15 MB PDF)

Text S2 **List of identified chemotaxis genes.**
(1.22 MB TXT)

## Abbreviations

CW: clockwise
RBS: ribosome-binding site
SD: Shine-Dalgarno
