# Supplementary material for: Role of Translational Coupling in Robustness of Bacterial Chemotaxis Pathway
Source: PLoS Biol. 2009 Aug 18;7(8):e1000171. doi: 10.1371/journal.pbio.1000171 (PMC2716512; doi:10.1371/journal.pbio.1000171)

Figure S1. Phylogenetic map of chemotaxis gene order in selected prokaryotes.

Receptor genes or *mcp* are indicated by „m“, *cheA* by „A“, *cheB* by „B“ etc. Minus indicates hypothetical protein of unknown function or protein unrelated to chemotaxis. Independent gene groups are separated by dots.

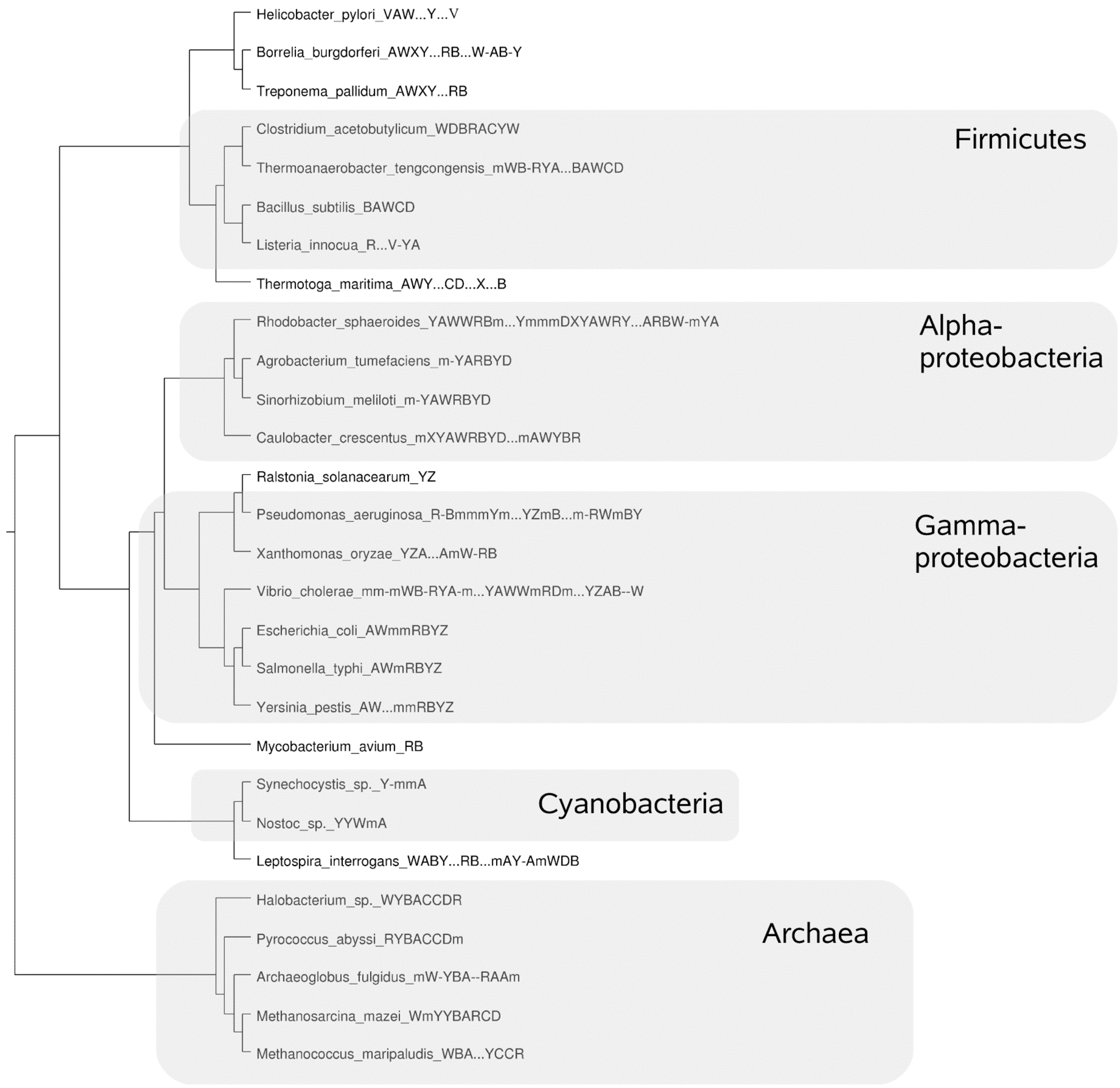

Supplement: Figure S1 — Phylogenetic map of chemotaxis gene order in selected prokaryotes. Order of chemotaxis genes in selected prokaryotes was mapped on the phylogenetic tree, constructed as described in Materials and Methods. Receptor genes or mcp are indicated by m, cheA by A, cheB by B, and so on. A minus sign (−) indicates hypothetical protein of unknown function or protein unrelated to chemotaxis. Independent gene groups are separated by dots. (0.44 MB PDF) [file pbio.1000171.s001.pdf]
