# Supplementary material for: Role of Translational Coupling in Robustness of Bacterial Chemotaxis Pathway
Source: PLoS Biol. 2009 Aug 18;7(8):e1000171. doi: 10.1371/journal.pbio.1000171 (PMC2716512; doi:10.1371/journal.pbio.1000171)
Supplement: Figure S2 — Pairwise distances between the most frequently neighboring chemotaxis genes over 527 genomes. Distance between neighboring chemotaxis genes was defined as the number of nucleotides between the last nucleotide of the stop codon of the upstream gene and the first nucleotide of start codon of the downstream gene. Intergenic distances were determined as described in Materials and Methods, and plotted as histograms. (0.47 MB PDF) [file pbio.1000171.s002.pdf]

Figure S2. Pairwise distances between the most frequently neighboring chemotaxis genes over 527 genomes.

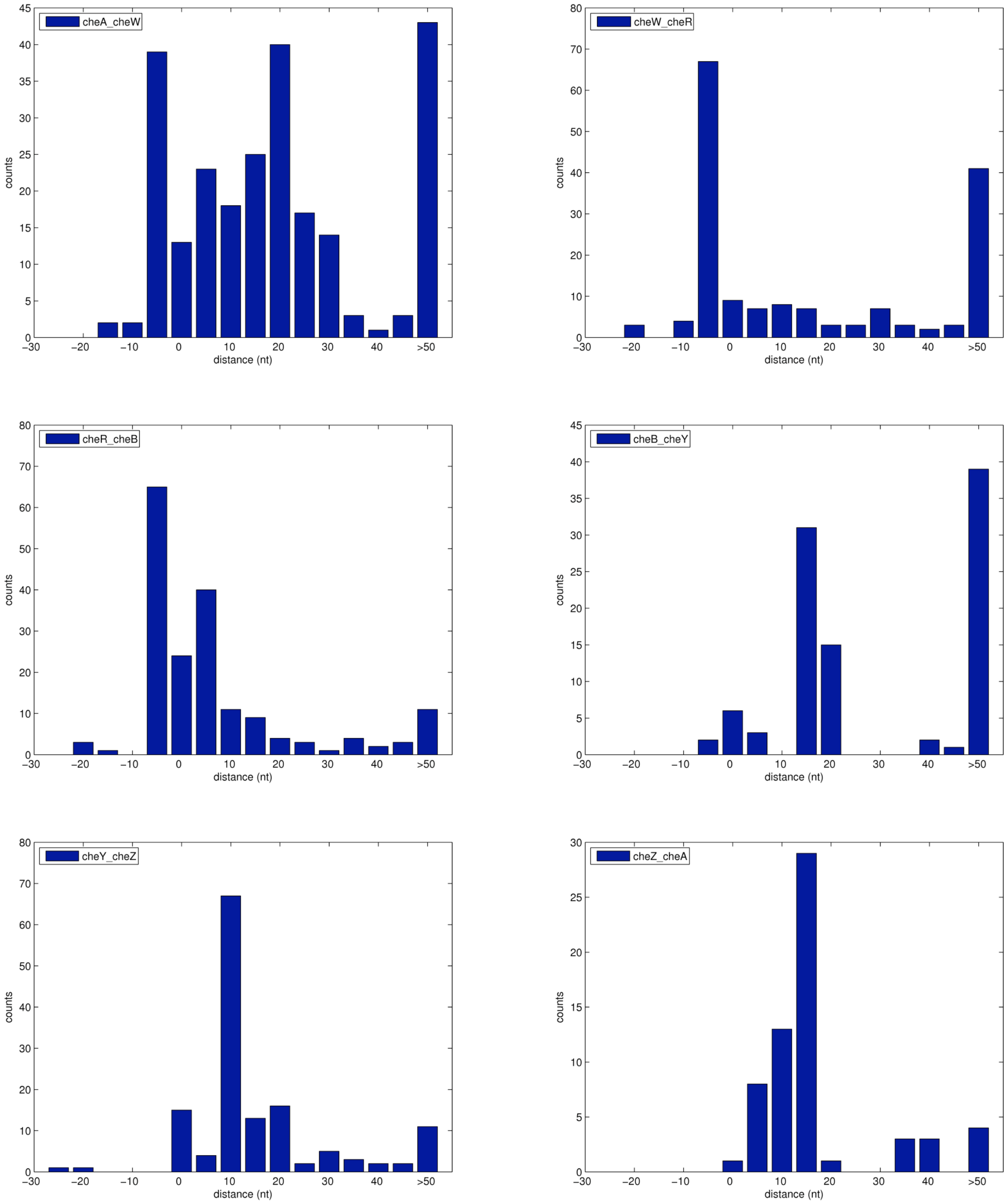

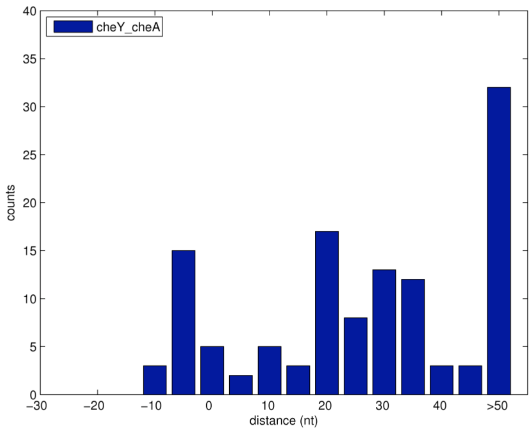
cheY_cheA
counts
distance (nt)
−30 −20 −10 0 10 20 30 40 >50
0 5 10 15 20 25 30 35 40

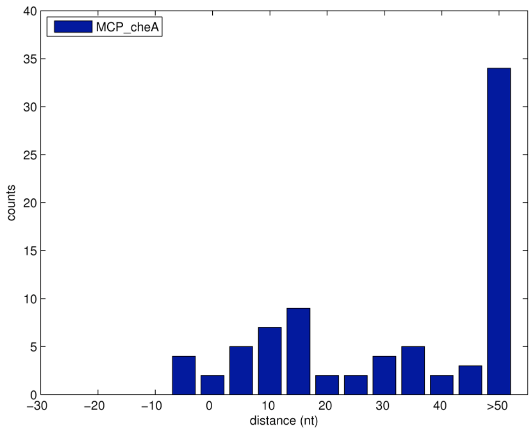
MCP_cheA
counts
distance (nt)
−30 −20 −10 0 10 20 30 40 >50
0 5 10 15 20 25 30 35 40

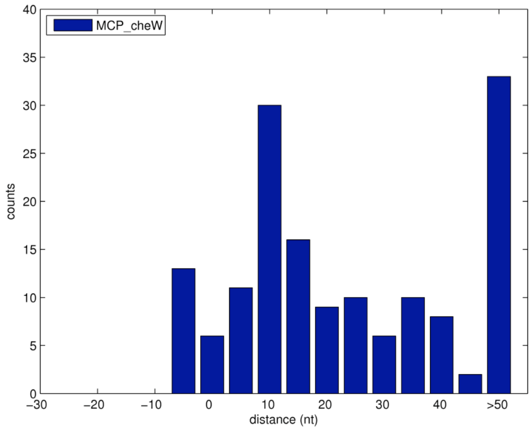
MCP_cheW
counts
distance (nt)
−30 −20 −10 0 10 20 30 40 >50
0 5 10 15 20 25 30 35 40

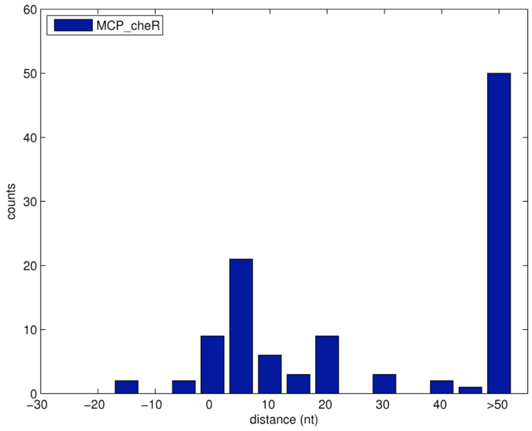
MCP_cheR
counts
distance (nt)
−30 −20 −10 0 10 20 30 40 >50
0 10 20 30 40 50 60

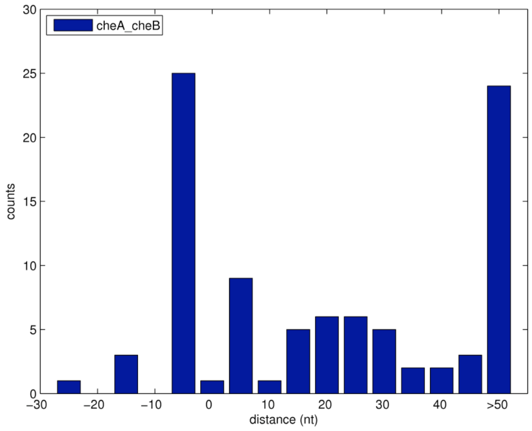
cheA_cheB
counts
distance (nt)
−30 −20 −10 0 10 20 30 40 >50
0 5 10 15 20 25 30

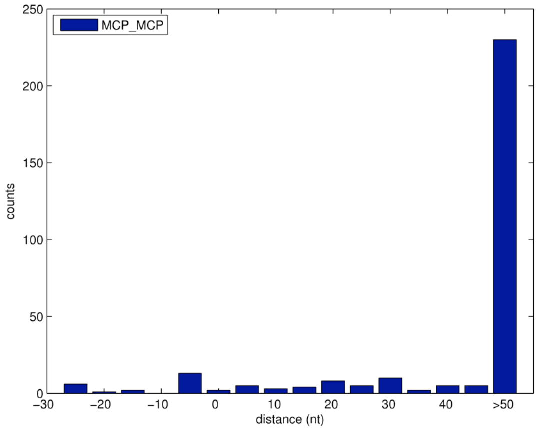
MCP_MCP
counts
distance (nt)
−30 −20 −10 0 10 20 30 40 >50
0 50 100 150 200 250

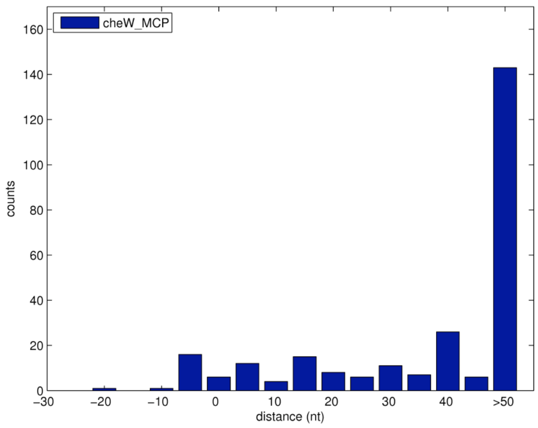
cheW_MCP
counts
distance (nt)
0
20
40
60
80
100
120
140
160
−30
−20
−10
0
10
20
30
40
>50
