# Supplementary material for: Role of Translational Coupling in Robustness of Bacterial Chemotaxis Pathway
Source: PLoS Biol. 2009 Aug 18;7(8):e1000171. doi: 10.1371/journal.pbio.1000171 (PMC2716512; doi:10.1371/journal.pbio.1000171)

Figure S3. Chemotactic selection for post-transcriptional coupling of CheY-YFP and CheZ-CFP at 10 µM IPTG induction.

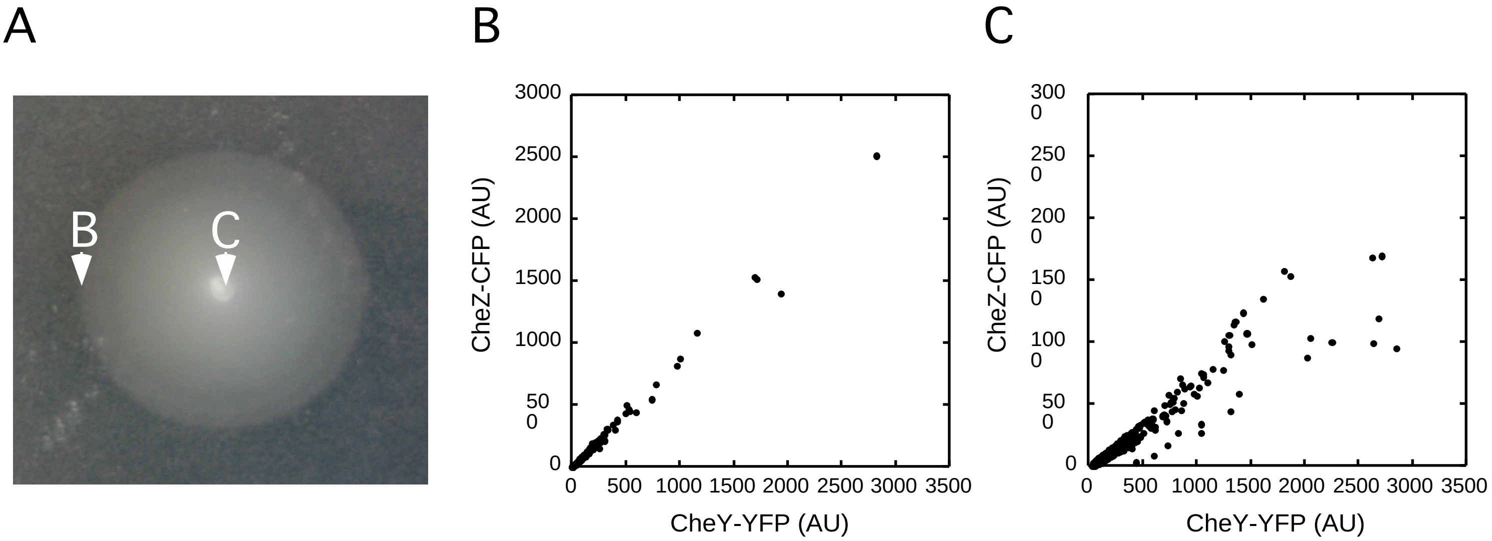

Supplement: Figure S3 — Chemotactic selection for posttranscriptional coupling of CheY-YFP and CheZ-CFP at 10 µM IPTG induction. (A) Chemotaxis-driven spreading of VS104 [Δ(cheYcheZ)]/pVS88 cells on soft agar (swarm) plates. (B and C) Scatter plots of single-cell levels of CheY-YFP and CheZ-CFP in cells taken from the edge (B) and from the middle (C) of the spreading colony. Relative concentrations of fluorescent proteins in individual cells were determined using fluorescence microscopy as described in Materials and Methods. See description of Figure 3 in the main text for more details. (0.62 MB PDF) [file pbio.1000171.s003.pdf]
