# Supplementary material for: Role of Translational Coupling in Robustness of Bacterial Chemotaxis Pathway
Source: PLoS Biol. 2009 Aug 18;7(8):e1000171. doi: 10.1371/journal.pbio.1000171 (PMC2716512; doi:10.1371/journal.pbio.1000171)
Supplement: Table S1 — Terms used for identification of chemotaxis genes. (0.07 MB DOC) [file pbio.1000171.s004.doc]

|  |
| --- |
|  |
|  |
|  |
|  |
|  |
|  |
|  |
|  |
|  |
|  |
|  |
|  |
|  |
|  |
|  |
|  |
|  |
|  |
|  |
|  |
|  |
|  |

**Table S1.** Terms used for identification of chemotaxis genes.

***Chemotaxis gene recognition by our program required its annotation to contain one of the „positive“ and no „negative“ terms listed below:***

| ***cheA*** |
| --- |
| Positive terms: cheA; chemotaxis histidine kinase |
|  |
| ***cheB*** |
| Positive terms: cheB; regulates chemotaxis by demethylation; chemotaxis(-specific) methylesterase; |
| Negative terms^a^: cheR; methyltransferase; methylates; methylase; histidine kinase |
|  |
| ***cheR*** |
| Positive terms: cheR; methylase of chemotaxis methyl-accepting protein; chemotaxis (protein) methyltransferase/methylase; MCP methyltransferase(,) CheR-type; |
| Negative terms: cheB; methylesterase; demethylation; pili; inhibition; CheV |
|  |
| ***cheY****^b^* |
| Positive terms: cheY; chemotaxis response regulator; |
|  |
| ***cheW****^b^* |
| Positive term: cheW |
| Negative term: histidine kinase |
|  |
| ***cheZ*** |
| Positive term: cheZ |
|  |
| ***mcp*** |
| Positive terms: mcp; chemotaxis sensory transducer; methyl-accepting chemotaxis; |
| Negative terms: methyltransferase; methylase; methylation; methylates; methylesterase; demethylation; histidine kinase; catalyzes; |

^a^Negative expressions indicate that gene function is ambiguous or related to another chemotaxis gene.

^b^The number of *cheY* and *cheW* genes in our analysis is probably overestimated, because many other proteins share homologous domains and negative patterns are difficult to define.
