# Supplementary material for: Role of Translational Coupling in Robustness of Bacterial Chemotaxis Pathway
Source: PLoS Biol. 2009 Aug 18;7(8):e1000171. doi: 10.1371/journal.pbio.1000171 (PMC2716512; doi:10.1371/journal.pbio.1000171)
Supplement: Table S2 — Pairwise occurrence of chemotaxis genes in 200 genomes containing cheZ . (0.05 MB DOC) [file pbio.1000171.s005.doc]

**Table S2.** Absolute frequencies^a^ of a pairwise occurrence of chemotaxis genes in 200 genomes containing *cheZ*.

|  | *cheA (346)* | | *cheW (610)* | | *cheR (347)* | | *cheB (343)* | | *cheY (482)* | | *cheZ (209)* | | *mcp^b^ (3392)* | |
| --- | --- | --- | --- | --- | --- | --- | --- | --- | --- | --- | --- | --- | --- | --- |
|  | left | right | left | right | left | right | left | right | left | right | left | right | left | right |
| *cheA* | 1.2 | 1.2 | **18.2** | 3.1 | <1 | <1 | **17.5** | <1 | <1 | **10.2** | <1 | **32.5** | 1.0 | <1 |
| *cheW* | 8.4 | **37.9** | 8.7 | 8.5 | **25.6** | **10.4** | 4.1 | <1 | 1.0 | 1.7 | 0 | 0 | **3.9** | **2.8** |
| *cheR* | <1 | 1.4 | 5.7 | **14.8** | <1 | <1 | **29.4** | 1.7 | 1.5 | 0 | 0 | 0 | <1 | **2.9** |
| *cheB* | <1 | **22.3** | <1 | 2.3 | 1.7 | **31.1** | <1 | <1 | **17.0** | <1 | <1 | 0 | <1 | <1 |
| *cheY* | **15.3** | <1 | 1.6 | <1 | 0 | 1.7 | <1 | **23.9** | 2.1 | 1.9 | **90.0** | 0 | <1 | <1 |
| *cheZ* | **17.9** | <1 | 0 | 0 | 0 | 0 | 0 | 0 | <1 | **27.4** | 0 | 0 | <1 | 0 |
| *mcp* | 6.1 | 9.8 | **13.4** | **16.7** | **32.0** | 0 | 1.2 | 1.2 | 2.7 | 1.5 | 0 | <1 | **3.9** | **3.7** |

^a^Absolute frequences were calculated as a number of gene occurrences in -1 (left neighbour) or +1 (right neighbour) positions relative to a reference gene, normalized by the total number of reference gene counts (shown in brackets). Strongest genomic coupling on each side (highest co-occurrence frequency) is marked in bold.

^b^Genes encoding chemoreceptors (methyl-accepting chemotaxis proteins).
