# Supplementary material for: Role of Translational Coupling in Robustness of Bacterial Chemotaxis Pathway
Source: PLoS Biol. 2009 Aug 18;7(8):e1000171. doi: 10.1371/journal.pbio.1000171 (PMC2716512; doi:10.1371/journal.pbio.1000171)
Supplement: Table S3 — Pairwise occurrence of chemotaxis genes in 327 genomes without cheZ . (0.05 MB DOC) [file pbio.1000171.s006.doc]

**Table S3.** Absolute frequencies^a^ of a pairwise occurrence of chemotaxis genes in 327 genomes without *cheZ*.

|  | *cheA (426)* | | *cheW (622)* | | *cheR (455)* | | *cheB (313)* | | *cheY (894)* | | *mcp^b^ (3128)* | |
| --- | --- | --- | --- | --- | --- | --- | --- | --- | --- | --- | --- | --- |
|  | left | right | left | right | left | right | left | right | left | right | left | right |
| *cheA* | 0.9 | 0.5 | **21.1** | 3.2 | 4.2 | 4.0 | **10.5** | **10.2** | 0.3 | **6.5** | 0.4 | 1.5 |
| *cheW* | 6.3 | **37.8** | 3.1 | 2.7 | **17.4** | 5.1 | 6.4 | 2.2 | **2.8** | **3.5** | **4.0** | **3.3** |
| *cheR* | 4.0 | 5.9 | 3.5 | **12.7** | 0.2 | 0.2 | **25.9** | **20.4** | 2.1 | 1.1 | 0.5 | 1.2 |
| *cheB* | 9.2 | **9.2** | 1.3 | 3.1 | **13.8** | **18.0** | 0.6 | 0.6 | **2.0** | 3.2 | 0.3 | 0.1 |
| *cheY* | **16.0** | 0.9 | 5.1 | 3.9 | 2.0 | 4.2 | 9.6 | 5.4 | 1.5 | 1.6 | 0.5 | 0.4 |
| *mcp* | **13.6** | 3.5 | **12.4** | **16.2** | 5.3 | 3.7 | 1.0 | 3.5 | 1.5 | 1.1 | **6.4** | **6.4** |

^a^Absolute frequences were calculated as a number of gene occurrences in -1 (left neighbour) or +1 (right neighbour) positions relative to a reference gene, normalized by the total number of reference gene counts (shown in brackets). Strongest genomic coupling on each side (highest co-occurrence frequency) is marked in bold.

^b^Genes encoding chemoreceptors (methyl-accepting chemotaxis proteins).
