# Supplementary material for: Role of Translational Coupling in Robustness of Bacterial Chemotaxis Pathway
Source: PLoS Biol. 2009 Aug 18;7(8):e1000171. doi: 10.1371/journal.pbio.1000171 (PMC2716512; doi:10.1371/journal.pbio.1000171)
Supplement: Text S1 — Mathematical model. (0.15 MB PDF) [file pbio.1000171.s007.pdf]

Text S1. Mathematical modelling

1 Modeling of protein-fluctuations and correlations

Consider a single copy of polycistronic mRNA coding for M different proteins. The gene content of the ith protein of this mRNA consists of N_i codons and is translated simultaneously by k_i ribosomes. The strength of the ribosome binding sites are determined by Michaelis-Menten constants, K_i^M. The fraction of codons occupied by ribosomes is given by $x_i = k_i/N_i$ and thus independent of the length of coding sequence. We denote the ribosome concentration by R and model stochastic accessibility $A_i = A_i(t, x_{i-1})$ of ith ribosome binding site by a random telegraph process, i.e. stochastic switching of the mRNA between only two states, an accessible and an inaccessible state. This state is assumed to be dependent on upstream translation efficiency, x_{i-1}. On the translational timescale the average fraction of codons occupied by ribosomes is then given by

$$x_i = \frac{k^{on}}{v} \frac{R}{K_i^M + R} \langle A_i(t, x_{i-1}) \rangle \tag{1}$$

with k^{on} the rate of initiation of translation and v the translational velocity, $k^{on} \leq v$. Here, $A_i(t, x_{i-1})$, is assumed to be a fast fluctuating variable on time scales of translation, such that A_i can be substituted by its average $\langle A_i \rangle$. We also assume in the following that translational coupling arises from a positive correlation of the codon occupation probability by ribosomes, x_i, between upstream genes. The rationale is that the higher the ribosome density on the upstream gene $i - 1$ the more ribosomes start translation of the gene, i, as a consequence of an increase in $\langle A_i(t, x_{i-1}) \rangle$. Changes in the initiation rate of the ith gene, $k^{on} \langle A_i(t, x_{i-1}) \rangle$, can be explained e.g. by changes in the mRNA secondary structure. This simple model is able to account for the experimental fact that an upstream gene with weak ribosome binding site can significantly influence translational efficiency of a downstream gene with strong ribosome binding site.

The assumption that translational coupling works predominantly in downstream direction leads to a change in translational rate of the ith gene, Δx_i, in response to an induced increase in ribosome density, Δx_{i-1}^{ind}, of the upstream gene with index $i - 1$. To linear order this response is given by

$$\frac{\Delta x_i}{x_i^0} = \alpha_{ii-1} \frac{\Delta x_{i-1}^{ind}}{x_{i-1}^0} \qquad \text{with} \quad \alpha_{ij} < 1 \tag{2}$$

with x_i^0 the average ribosome density of the population and $\Delta\, x_i^{ind}$ the external induced deviations from the native ribosome-densities, e.g. by mutants with stronger ribosome binding sites. The coupling constants depend on changes relative to the average ribosome density but not their absolute values to ensure coupling between weakly and strongly translated genes. The general coupling between any two genes is given by

$$\Delta x_i = A_{ij} \Delta x_j^{ind} \quad . \tag{3}$$

On time scales of the mean protein translation time we expect this linear response relation to hold also for stochastic fluctuation in ribosome density such that Δx_j^{ind} can be substituted by $\Delta x_j = x_j - x_j^0$. For equal response coefficients $\alpha_{i,i-1} \approx \alpha$ as observed in experiments (this work) the structure of the response matrix $\mathbf{A}$ reads

$$\mathbf{A} = \begin{pmatrix} 1 & 0 & \multicolumn{3}{c}{\dots\dots\dots\dots\dots\dots\dots\dots\dots\dots} & 0 \\ \alpha \frac{x_2^0}{x_1^0} & 1 & 0 & \multicolumn{2}{c}{\dots\dots\dots\dots\dots\dots\dots} & 0 \\ \alpha^2 \frac{x_3^0}{x_1^0} & \alpha \frac{x_3^0}{x_2^0} & 1 & 0 & \dots\dots\dots & 0 \\ \vdots & \vdots & \vdots & \vdots & & \vdots \\ \alpha^{N-1} \frac{x_N^0}{x_1^0} & \alpha^{N-2} \frac{x_N^0}{x_2^0} & \alpha^{N-3} \frac{x_N^0}{x_3^0} & \alpha^{N-4} \frac{x_N^0}{x_4^0} & \dots \quad \alpha \frac{x_N^0}{x_{N-1}^0} & 1 \end{pmatrix} . \tag{4}$$

The matrix reflects that changes in ribosome density N genes upstream of a given gene result in an α^N fold change in translational efficiency on average. As the response coefficient is significantly smaller than one ($\alpha \approx 0.25$) effects on translational efficiency arise predominantly from adjacent genes.

1.1 Fluctuations in ribosome density due to stochastic binding

In the following we try to estimate the fluctuations in the time-averaged ribosome densities x_i originating from stochastic independent binding events of ribosomes. The stationary probability for the number k_i of ribosomes on the gene i is in this case given by a binomial distribution

$$p(k_i) = \binom{N_i}{k_i} ({x^0}_i)^{k_i} \left(1 - x_i^0\right)^{N_i - k_i} . \tag{5}$$

As the ribosome density is in general small, $x_i^0 \ll 1$ and $\langle k_i \rangle \; = x_i^0 N_i \ll N_i$ Eq.(5) follows a Poisson distribution to good approximation

$$p(k_i) \approx \frac{\langle k_i \rangle^{k_i}}{k_i!} e^{-\langle k_i \rangle} \tag{6}$$

that in turn can approximated by a Gaussian distribution for $\langle k_i \rangle > 10$

$$p(x_i) = \frac{1}{\sqrt{2\pi\sigma_i^2}} \exp\left[-\frac{\left(x_i - x_i^0\right)^2}{2\sigma_i^2} \right], \quad \sigma_i^2 = \frac{x_i^0}{N_i}. \tag{7}$$

By Eq. (3) the ribosome densities, x_i, depend on the upstream ribosome densities $x_i = x_i(x_{i-1})$. Translational coupling thus leads to a joint probability distribution of the form

$$p(\mathbf{x}) = \frac{1}{(2\pi)^{\frac{N}{2}} \sqrt{\det(\boldsymbol{C})}} \exp\left[-\frac{1}{2}\Delta\,\mathbf{x}^T \cdot \boldsymbol{C}^{-1} \cdot \Delta\,\mathbf{x}\right], \tag{8}$$

with the covariance matrix

$$\boldsymbol{C} = \mathbf{A} \cdot \mathrm{diag}(\sigma_1^2, \sigma_2^2, \ldots, \sigma_N^2) \cdot \mathbf{A}^T. \tag{9}$$

1.2 Protein copy numbers

On the translation time scale the dynamics of the protein copy number $P_i(t)$ can be captured by the simple differential equation

$$\partial_t P_i(t) = v\left(x_i^0 + \Delta x_i(t)\right) - \gamma P_i(t). \tag{10}$$

Here v denotes the translational speed and γ the dilution rate given by $\gamma = \ln(2)/\tau$, with τ being the generation time. We separate the mean and fluctuations using $x_i = x_i^0 + \Delta x_i(t)$, with $\langle \Delta x_i(t)\rangle = 0$. We assume further that the correlation time $1/\beta$ is about the same for all genes, i.e. $\langle \Delta x_i(t)\Delta x_j(t')\rangle = C_{ij}e^{-\beta|t-t'|}$ for all i, j. The solution of Eq. (10) yields

$$P_i(t) = \int_{-\infty}^{t} e^{-\gamma(t-t')} v\left(x_i^0 + \Delta x_i(t')\right) dt'. \tag{11}$$

Since integration is a linear operation the distribution for P_i is also Gaussian. The expectation value of P_i corresponds to the population average and is given by

$$\langle P_i(t)\rangle = \int_{-\infty}^{t} e^{-\gamma(t-t')} v x_i^0 dt' = \frac{v}{\gamma} x_i^0. \tag{12}$$

Within our model the number of proteins being expressed from one mRNA only depends on the strength of its ribosome binding site and the translation initiation rate but not on the translational velocity. The correlation between the ith and jth gene product, P_i and P_j, is given by

$$\begin{aligned}
\langle P_i(t)P_j(t)\rangle &= \left\langle \int_{-\infty}^{t} e^{-\gamma(t-t')} v\left(x_i^0 + \Delta x_i(t')\right) dt' \int_{-\infty}^{t} e^{-\gamma(t-t'')} v\left(x_j^0 + \Delta x_j(t'')\right) dt'' \right\rangle \\
&= \left\langle \int_{-\infty}^{t} dt' \int_{-\infty}^{t} dt'' e^{-\gamma(2t-t'-t'')} v^2 \left[x_i^0 x_j^0 + x_j^0 \Delta x_i(t') + x_i^0 \Delta x_j(t'') + \Delta x_i(t')\Delta x_j(t'')\right] \right\rangle \\
&= \langle P_i(t)\rangle\langle P_j(t)\rangle + v^2 \int_{-\infty}^{t} dt' \int_{-\infty}^{t} dt'' e^{-\gamma(2t-t'-t'')} \langle \Delta x_i(t')\Delta x_j(t'')\rangle \\
&= \langle P_i(t)\rangle\langle P_j(t)\rangle + v^2 C_{ij} \int_{-\infty}^{t} dt' \int_{-\infty}^{t} dt'' e^{-\gamma(2t-t'-t'')} e^{-\beta|t'-t''|} \\
&= \langle P_i(t)\rangle\langle P_j(t)\rangle + v^2 C_{ij} \int_{-\infty}^{t} dt' \left[\frac{e^{-2\gamma(t-t')}}{\beta+\gamma} + \frac{e^{-2\gamma(t-t')}}{\beta-\gamma} - \frac{e^{-(\beta+\gamma)(t-t')}}{\beta-\gamma}\right]
\end{aligned}$$

$$= \langle P_i(t)\rangle\langle P_j(t)\rangle + \frac{v^2 C_{ij}}{\gamma(\beta+\gamma)}. \tag{13}$$

Since we assumed equal correlation times, the covariance matrix is given by

$$\Xi_{ij} = \langle P_i(t)P_j(t)\rangle - \langle P_i(t)\rangle\langle P_j(t)\rangle = \frac{v^2}{\gamma(\gamma+\beta)} C_{ij}. \tag{14}$$

The stationary probability distribution for the copy numbers P_i finally reads

$$p(\mathbf{P}) = \frac{1}{(2\pi)^{\frac{N}{2}}\sqrt{\det(\Xi)}} \exp\left[-\frac{1}{2}(\mathbf{P} - \langle\mathbf{P}\rangle)^T \cdot \Xi^{-1} \cdot (\mathbf{P} - \langle\mathbf{P}\rangle)\right]. \tag{15}$$

1.3 Intrinsic Noise due to stochasticity in translation

The timescale for fluctuations in ribosome density on gene i is of the order the time it takes to translate a protein, thus $\beta \approx v/N \approx 0.1\,\mathrm{s}^{-1}$, where we assumed a translational speed of $v = 20\,\mathrm{aa}$ per second and a hypothetical gene length of $N = 200\,\mathrm{aa}$.

The variance of fluctuations in protein copy number can be calculated by using (14) and the approximation $\frac{1}{\gamma(\beta+\gamma)} \approx \frac{1}{\gamma\beta}$. Hence, for the case without translational coupling we obtain for the variance of gene i

$$\sigma^2_{P_i} = \frac{v}{\beta}\frac{\langle P_i\rangle}{N_i} \approx \langle P_i\rangle \tag{16}$$

and for the relative fluctuations

$$\eta_i = \frac{\sigma_{P_i}}{\langle P_i\rangle} \approx \frac{1}{\sqrt{\langle P_i\rangle}}. \tag{17}$$

So far we have considered the situation where only one mRNA is present in the cell at every instant of time. If the same amount of protein, $\langle P_i\rangle$, is synthesized by m mRNAs the noise in proteins synthesized exclusively from the k-th mRNA is given by $\sigma^2_{P_i k} = \langle P_i\rangle/m$. However, the variance of the total protein copy number in the cell is given again by $\sigma^2_{P_i} = \sum_{k=1}^{m}\sigma^2_{P_i k} = \langle P_i\rangle$ because of stochastic independence of the translational events on each mRNA. The variations in protein copy number of CheY and CheZ from a dicistronic plasmid should reflect the translational noise as proteins are diluted by cell division. However, the measured standard deviation over mean in protein copy number of ≈ 0.2 is far beyond the expected value from our analysis $\sigma_{P_Z}/\langle P_Z\rangle \approx 1/\sqrt{2500} = 0.02$. This gives rise to the hypothesis that other regulatory elements determine translational noise and not stochastic ribosome binding and translation initiation events.

1.4 Protein concentrations

Actually we are interested in the protein concentration c_i, hence we have to divide the copy number P_i by the cell volume V_c,

$$c_i = \frac{P_i}{V_c}. \tag{18}$$

In our simulations the stochastic variables c_i are generated by the expression

$$\mathbf{c} = \xi^{ex}\langle\mathbf{c}\rangle + \sqrt{\xi^{ex}}\,\nu\,\mathbf{A}\cdot\mathrm{diag}\left(\langle c_1\rangle\eta_1, \langle c_2\rangle\eta_2, \ldots, \langle c_N\rangle\eta_N\right)\cdot\boldsymbol{\xi}^{in}, \tag{19}$$

with

$$\nu\eta_i = \frac{\sigma_{P_i}}{\langle P_i\rangle}, \quad \langle c_i\rangle = \frac{\langle P_i\rangle}{V_c}. \tag{20}$$

The parameter ν controls the amount of intrinsic noise and the η_i's specify the differences in relative noise. Eqs. (12) and (20) were used to substitute x_i^0 in Eq. (4).

The log-normal distributed variables ξ^{ex} are obtained from

$$\xi^{ex} = N\exp\left[\omega\xi\ln(10)\right], \quad \omega = 0.2 \tag{21}$$

with N chosen such that $\langle\xi^{ex}\rangle = 1$. The variables ξ and the vector $\boldsymbol{\xi}^{in}$ are independent normal distributed stochastic variables with mean zero and variance one. We use additionally a cutoff for low expression levels that results in ignoring values of ξ^{ex} below 0.5. The physical reason for the existence of a cutoff is that all proteins of the chemotaxis pathway strongly localize at the receptor clusters and get distributed to the daughter cells roughly proportional to the large receptor clusters at the cell poles. Note that there still exists a significant amount of cells in the *in silico* population with protein expression levels below half their mean level due to the intrinsic noise contribution.

2 Ranking of Gene Permutations

E.coli performs chemotaxis by a biased random walk. Swimming runs are interrupted by tumbling events, i.e. events that partially randomize the direction the bacterium is moving. By tuning the frequency of tumbling events in response to changing attractant concentrations the bacteria swims into favorable directions and avoids swimming into unfavorable ones. Swimming is accomplished by a concerted rotation of the flagellar motors in the counter-clockwise sense (CCW), whereas clockwise (CW) rotation of the motors leads to tumbling. The CW bias is controlled by the concentration of free phosphorylated CheY and follows a steep response curve with a Hill coefficient of ten [1]. It was shown that maintaining a certain CW bias in the adapted state is a reliable measure for chemotactic performance [2].

In our setup, we solve the stationary state equations (see section 3.4) governing the chemotaxis pathway subject to different total concentrations of the involved proteins as determined by stochastic gene expression (19). The cell-to-cell variations of the protein concentrations lead to a distribution in the adapted CheYp level within a population and hence to a distribution in the CW bias, calculated by

$$\text{CW-bias} = \frac{(Yp)^{10}}{(K_h)^{10} + (Yp)^{10}}. \tag{22}$$

Within the Meche operon, all 24 permutations of the gene order of the chemotaxis proteins CheR, CheB, CheY and CheZ are simulated according to Eq. (19).

For each sample consisting of 10^5 cells the value of K_h was determined such that the physiological value of the average CW bias is simply $\langle \text{CW-bias} \rangle = 0.2$. The standard deviation in CW bias is a measure of the chemotaxis efficiency within a population. We therefore rank the different gene permutations according to the standard deviation in CW bias.

Additionally, we performed simulations with proteins that are perfectly coupled in gene expression. Perfect coupling between concentrations of any two proteins, c_1 and c_2, results from the relation

$$c_2 = \langle c_2 \rangle \times \frac{c_1}{\langle c_1 \rangle}, \tag{23}$$

with c_1 a stochastic concentration in an otherwise uncoupled random fluctuating background. The stochastic variables were taken from a multi-Gaussian distribution (19) with $\mathbf{A} = \mathbf{1}$ being the unit matrix, $\omega = 0$, $\nu = 0.05$ and $\eta_i = 1$.

The results are shown in Fig. 4A. The dotted line in this bar-plot shows the standard deviation in CW bias without any coupling. Most of the pairings lead to a decrease of the standard deviation, but interestingly two of them lead to worse chemotactic performance. We can conclude that there are favorable and unfavorable pairings of chemotaxis genes depending on whether they decrease or increase the standard deviation in CW bias.

This result enables us to understand the ranking of the 24 permutation, shown in Fig. 4B. The noise was generated by using a coupling parameter $\alpha = 0.25$, $\omega = 0$, $\nu = 0.05$ and $\eta_i = 1$. The zero value for the extrinsic noise, $\omega = 0$, was chosen, since additional

extrinsic noise has no effect on the ranking of permutations as this is a robust system property as shown in [2].

Three blocks of permutation can be identified in Fig. 4B. They differ in respect to the number of favorable pairings. The first block reaching from *RZYB* to *ZRBY* consists of permutation with no unfavorable pairings. The second and third block, reaching from *BRYZ* to *RZBY* and *RYZB* to *YRBZ*, are made up of permutations with one and two unfavorable pairings, respectively. Apart from this gene order does not matter in Fig. 4B, as can be seen by the fact that each permutation and counterpart arising from reflection-symmetry perform within statistical fluctuations equally well. Explanation of the ranking order on grounds of favorable an unfavorable pairings is possible, as in our model correlations in expression levels decay exponentially fast

$$\varrho(c_i, c_j) \sim \alpha^{|i-j|} + \mathcal{O}(\alpha^{|i-j|+2}). \tag{24}$$

The ranking order changes if we assume a higher relative noise for CheR and CheB, i.e. $\eta_R = \eta_B = 1.5$, as shown in Fig. 4D. In this case order of pairings now matters, as for example *BYRZ* and *ZRYB*, previously ranked equally, now occupy different places. The permutation *BYRZ* is ranked much higher compared to *ZRYB*. The reason for this is of course the difference in noise levels, leading to distinct correlation coefficients depending on the order of pairings

$$\varrho(c_{i-1}, c_i) = \alpha \frac{\eta_{i-1}}{\eta_i} + \mathcal{O}(\alpha^3). \tag{25}$$

This can intuitively be understood if one considers the case where one of the proteins is expressed with no noise at all. First, assume the noisy protein in the first place. Due to the coupling, the non-fluctuating ones is able to follow the translational fluctuations of the first protein closely, however this is not the case if we reverse the order. In this case the second protein is still fluctuating and therefore correlations are smaller compared to the first case.

Given the expression (25) we see that depending on the correlation coefficient pairings are weighted differently. Hence the effect of beneficial pairings can be amplified, whereas the effect of unfavorable pairings can be attenuated. Consider our example from above, i.e *BYRZ* and *ZRYB*. For the first permutation the correlation coefficients, calculated by (25) with $\eta_R = \eta_B = 1.5$ and $\eta_Y = \eta_Z = 1$ read

$$\varrho(\mathrm{B}, \mathrm{Y}) \sim 0.375 \tag{26}$$
$$\varrho(\mathrm{Y}, \mathrm{R}) \sim 0.167 \tag{27}$$
$$\varrho(\mathrm{R}, \mathrm{Z}) \sim 0.375. \tag{28}$$

In contrast, the correlation coefficients for *ZRYB* are

$$\varrho(\mathrm{Z}, \mathrm{R}) \sim 0.167 \tag{29}$$
$$\varrho(\mathrm{R}, \mathrm{Y}) \sim 0.375 \tag{30}$$
$$\varrho(\mathrm{Y}, \mathrm{B}) \sim 0.167. \tag{31}$$

Comparing these numbers, we see that in the permutation *BYRZ* the favorable pairings *BY* and *RZ* are stronger weighted than the unfavorable pairing *YR*. For the permutation *ZRYB* it is exactly the other way round, thus it is ranked much lower.

In addition to the gene order effect, the simple pattern of blocks formed by the permutations with a given number of unfavorable pairings has vanished, the permutation *BYRZ* is enclosed by *RZYB* and *YZRB*. The two latter one with only favorable pairings in contrast to the former with one unfavorable pair *YR*. Taking the other relevant correlation coefficients into account

$$\varrho(\mathrm{Z},\mathrm{Y}) \sim \varrho(\mathrm{Y},\mathrm{Z}) \sim \varrho(\mathrm{R},\mathrm{B}) \sim 0.25. \tag{32}$$

we learn that only one of the pairings, i.e. *RZ* is weighted by a higher correlation coefficient of ~ 0.375, whereas the other pairings have a correlation coefficient of ~ 0.25 or even ~ 0.167. Hence the larger correlation in the case of *BYRZ* of the beneficial pairings and the attenuation of the bad pair seems to be sufficient to make it equivalent to permutations with only good pairings.

In summary we can conclude that if there are differences in noise level between the proteins, the ranking of gene order is altered by the relation (25). Attenuation of disadvantageous and amplification of beneficial pairings have to be taken into account to understand the effects on chemotactical performance.

An additional effect which could be important for the determination of the gene-order is the propagation of noise. Due to translational coupling the standard deviations of proteins at the backmost positions within the operon become larger. In our model, however, this effect is only of second order in α.

Since the CW bias is determined by the free Yp level within the cell, we will do a linear noise approximation of the free Yp concentration with respect to fluctuations of the total concentrations of CheA, CheY, CheZ, CheR and CheB. If the fluctuations, denoted by $\delta A^T, \delta Y^T, \delta Z^T, \delta R^T$ and δB^T, are sufficiently small, it is possible to determine the ranking of permutations by this expansion.

The expansion of Yp up to linear order is given by

$$\delta Yp = \frac{\partial Yp}{\partial A^T}\delta A^T + \frac{\partial Yp}{\partial Y^T}\delta Y^T + \frac{\partial Yp}{\partial Z^T}\delta Z^T + \frac{\partial Yp}{\partial R^T}\delta R^T + \frac{\partial Yp}{\partial B^T}\delta B^T. \tag{33}$$

Calculating the variance of Yp implies

$$\langle\langle Yp \rangle\rangle = \langle(\delta Yp)^2\rangle. \tag{34}$$

We numerate the proteins according to their position on the operon and use c_i to indicate the total concentration of the ith protein (compare section 1). Furthermore, CheA concentration is denoted by c_0 and there is no translational coupling between the kinase and the remaining chemotaxis proteins. Thus, we get for the variance up to linear order in the fluctuating proteins

$$\langle(\delta Yp)^2\rangle = \sum_{i=0}^{4} \left(\frac{\partial Yp}{\partial c_i}\right)^2 \langle(\delta c_i)^2\rangle + \sum_{\substack{i,j=1 \\ i\neq j}}^{4} \frac{\partial Yp}{\partial c_i}\frac{\partial Yp}{\partial c_j}\langle \delta c_i \delta c_j\rangle. \tag{35}$$

All derivatives are performed at the average wild-type protein concentration. The first sum refers to the noise in the system and the second to the correlations. We can now separate the influences on the variance of Yp. The derivatives give the response of the biochemical network to the perturbations in protein concentrations, whereas $\langle(\delta c_i)^2\rangle$ and $\langle\delta c_i \delta c_j\rangle$ reflect the statistical properties of the gene expression process.

Using our model for gene expression noise (19) in absence of extrinsic noise, $\omega = 0$, and neglecting terms in quadratic or higher order in the coupling parameter α, we only get correlation between next neighbors,

$$\langle(\delta Yp)^2\rangle = \sum_{i=0}^{4}\left(\frac{\partial Yp}{\partial c_i}\right)^2 \langle(\delta c_i)^2\rangle + 2\sum_{i=2}^{4}\frac{\partial Yp}{\partial c_{i-1}}\frac{\partial Yp}{\partial c_i}\langle\delta c_{i-1}\delta c_i\rangle + \mathcal{O}(\alpha^2), \tag{36}$$

and the averages in this approximation read

$$\langle(\delta c_i)^2\rangle = \nu^2\eta_i^2\langle c_i\rangle^2 + \mathcal{O}(\alpha^2) \tag{37}$$

$$\langle\delta c_{i-1}\delta c_i\rangle = \alpha\nu^2\eta_{i-1}^2\langle c_{i-1}\rangle\langle c_i\rangle + \mathcal{O}(\alpha^3). \tag{38}$$

Here, we assumed that there is no extrinsic noise in the system, as taking it into account does not change the ranking of permutations, i.e. the effects of translational coupling. Extrinsic noise just adds to each of the normalized covariances $covar(c_i, c_j)/(\langle c_i\rangle\langle c_j\rangle)$ the variance of extrinsic noise and therefore does not lead to a differential ranking.

This approximation is sufficient to determine the ranking of the different permutations. Using $\sqrt{\langle(\delta Yp^2)\rangle}$ as given by Eq. (36), we recover essentially the same results as from the simulation. The derivatives were calculated numerically by using the model introduced in section 3.4.

Depending on the sign of the product $\frac{\partial Yp}{\partial c_{i-1}}\frac{\partial Yp}{\partial c_i}$, the correlations lead to an in- or decrease of the standard deviation of Yp, since the quadratic terms are always larger than zero. Independent of model details we can assume

$$\frac{\partial Yp}{\partial Y^T} > 0, \qquad \frac{\partial Yp}{\partial Z^T} < 0 \tag{39}$$

$$\frac{\partial Yp}{\partial R^T} > 0, \qquad \frac{\partial Yp}{\partial B^T} < 0. \tag{40}$$

Increasing the total CheY concentration means there is more CheY that can be phosphorylated. On the contrary more phosphatase CheZ in the system implies a decreasing Yp level. The enzymes CheR and CheB regulate the methylation of receptors and therefore the steady state receptor-activity, which affects the level of phosphorylated CheY. Increasing the CheR level leads to an increase in kinase activity, whereas increasing CheB concentration reduces kinase activity. Hence we concluded that

$$YZ \quad RB \quad YB \quad ZR \tag{41}$$

are favorable pairings, since the corresponding products of derivatives are negative. In contrast

$$YR \quad ZB \tag{42}$$

are unfavorable, as the product of derivatives is positive. Thus we recover the result from the analysis of pairwise coupling.

If all proteins show the same noise-level, i.e. η_i is the same for all, the order of pairs does not matter. However, with different noise level the correlation is stronger if the larger fluctuating protein is in the first place, i.e. $\eta_{i-1} > \eta_i$, since only η_{i-1} shows up in expression (38) for the correlation. Therefore the impact of favorable pairings can be amplified, those of unfavorable ones, both compared to the overall noise level, can be attenuated.

3 Model

3.1 Receptor methylation

The mathematical description for the methylation and phosphorylation kinetics of chemotaxis pathway follows mass action kinetics. As the output value of interest is the free equilibrated concentration of phosphorylated CheY, the details of transient methylation kinetics of the adaption machinery is of no concern here. However, methylation still determines the probability to find an allosteric receptor cluster in an active state, p_A,

$$p_A = \frac{k_R[RT]}{k_R[RT] + k_{Bp}[BpT]} \tag{43}$$

with receptor bound methyltransferase concentration $[RT]$ and receptor bound methylesterase concentration $[BpT]$, with Bp the phosphorylated form of CheB and rate constants $k_{Bp} \gg k_R$. Here, T denotes the concentration of functional receptors that are accessible for CheB and CheR. Note that the functional form of Eq.(43) assumes that CheR methylates only receptors in inactive state whereas CheB demethylates only active receptors. This assumption is not crucial as both *in vitro* and *in vivo* measurements of kinase activity show that only a small fraction of receptors are activated in the adapted state implying $k_R[RT] \ll k_{Bp}[BpT]$. The appearance of CheR concentration in the denominator of Eq.(43) due to a receptor activity dependent methylation results only a minor correction.

In vivo experiments show strong localization of phosphorylated CheB at the receptors but not of non-phosphorylated CheB. Therefore the receptor bound fraction of CheBp, $[BpT]$, follows

$$[BpT] \approx Bp. \tag{44}$$

We furthermore assume that also the total amount of available CheR is bound to the receptors, hence

$$[RT] \approx R^T. \tag{45}$$

3.2 Phosphorylation of response regulator CheY

The response regulator CheY is phosphorylated by the kinase CheA. This kinase consists of several domains, of which the P_2 domain binds the response regulator and the P_1 domain phosphorylates it. Additionally, phosphorylation takes place by a bimolecular reaction between CheY and the P_1 domain. The phosphorylation of CheA itself depends on the activity of the receptor complexes, denoted by p_A. We distinguish between different states of the kinase CheA, which is indicated by the notation

$$A[P_2 \cdot |P_1 \cdot]. \tag{46}$$

The P_2 domain can be occupied by an un-/phopsphorylated response regulator, CheY (Y, Yp) and CheB (B, Bp), respectively, conditional upon the phosphorylation state of the P_1 domain. The full phosphorylation reaction scheme therefore reads

$$
\begin{array}{ccccccc}
 & & & & A[P_2Yp|P_1p] & \underset{k_Y^4}{\overset{k_Y^5}{\rightleftharpoons}} & Yp + A[P_2|P_1p] \\
 & & & & k_Ap_A \Big\uparrow\Big\downarrow k_YY & & k_Ap_A \Big\uparrow\Big\downarrow k_YY \\
Y + A[P_2|P_1p] & \underset{k_Y^2}{\overset{k_Y^1}{\rightleftharpoons}} & A[P_2Y|P_1p] & \xrightarrow{k_Y^3} & A[P_2Yp|P_1] & \underset{k_Y^4}{\overset{k_Y^5}{\rightleftharpoons}} & Yp + A[P_2|P_1] \\
k_Ap_A \Big\uparrow\Big\downarrow k_YY & & k_Ap_A \Big\uparrow\Big\downarrow k_YY & & & & \\
Y + A[P_2|P_1] & \underset{k_Y^2}{\overset{k_Y^1}{\rightleftharpoons}} & A[P_2Y|P_1] & & & &
\end{array}
\tag{47}
$$

We omitted the interaction with the response regulator CheB for the sake of clarity.

The phosphorylated CheY is dephosphorylated by the phosphatase CheZ (Z). A complex $[YpZ]$ is formed which can lead to dephosphorylation. This mechanism can be depicted by the reaction scheme

$$Yp + Z \underset{k_Z^2}{\overset{k_Z^1}{\rightleftharpoons}} [YpZ] \xrightarrow{k_Z^3} Y + Z + p. \tag{48}$$

The full set of equations describing phosphorylation and dephosphorylation according to the reaction schemes (47) and (48) reads

$$
\begin{aligned}
\frac{d}{dt}Y &= -k_Y^1Y(A[P_2|P_1] + A[P_2|P_1p]) + k_Y^2(A[P_2Y|P_1] + A[P_2Y|P_1p]) \\
&\quad - k_YY(A[P_2|P_1p] + A[P_2Yp|P_1p] + A[P_2Y|P_1p]) + k_Z^3[YpZ] \qquad (49) \\
\frac{d}{dt}Yp &= k_Y^5(A[P_2Yp|P_1p] + A[P_2Yp|P_1]) - k_Y^4Yp(A[P_2|P_1] + A[P_2|P_1p]) \\
&\quad + k_YY(A[P_2|P_1p] + A[P_2Yp|P_1p] + A[P_2Y|P_1p]) - k_Z^1YpZ + k_Z^2[YpZ] \qquad (50) \\
\frac{d}{dt}[YpZ] &= k_Z^1YpZ - (k_Z^2 + k_Z^3)[YpZ] \qquad (51) \\
\frac{d}{dt}Z &= -k_Z^1YpZ + (k_Z^2 + k_Z^3)[YpZ] \qquad (52) \\
\frac{d}{dt}A[P_2|P_1] &= -k_Ap_AA[P_2|P_1] + k_YYA[P_2|P_1p] - k_Y^1YA[P_2|P_1] \\
&\quad + k_Y^2A[P_2Y|P_1] + k_Y^5A[P_2Yp|P_1] - k_Y^4YpA[P_2|P_1] \qquad (53) \\
\frac{d}{dt}A[P_2|P_1p] &= k_Ap_AA[P_2|P_1] - k_YYA[P_2|P_1p] - k_Y^1YA[P_2|P_1p] \\
&\quad + k_Y^2A[P_2Y|P_1p] + k_Y^5A[P_2Yp|P_1p] - k_Y^4YpA[P_2|P_1p] \qquad (54) \\
\frac{d}{dt}A[P_2Y|P_1] &= k_Y^1YA[P_2|P_1] - k_Y^2A[P_2Y|P_1] - k_Ap_AA[P_2Y|P_1] + k_YYA[P_2Y|P_1p] \qquad (55)
\end{aligned}
$$

$$\frac{d}{dt}A[P_2Y|P_1p] = k_Y^1 YA[P_2|P_1p] - k_Y^2 A[P_2Y|P_1p] + k_A p_A A[P_2Y|P_1] \\ - k_Y^3 A[P_2Y|P_1p] - k_Y YA[P_2Y|P_1p] \quad (56)$$

$$\frac{d}{dt}A[P_2Yp|P_1] = k_Y^3 A[P_2Y|P_1p] - k_A p_A A[P_2Yp|P_1] + k_Y YA[P_2Yp|P_1p] \\ - k_Y^5 A[P_2Yp|P_1] + k_Y^4 YpA[P_2|P_1] \quad (57)$$

$$\frac{d}{dt}A[P_2Yp|P_1p] = k_A p_A A[P_2Yp|P_1] - k_Y YA[P_2Yp|P_1p] - k_Y^5 A[P_2Yp|P_1p] + k_Y^4 YpA[P_2|P_1p] \quad (58)$$

Due to the small amount of CheB, i.e. $B^T \ll A^T$ and $B^T \ll Y^T$, all interactions of CheB with CheA are neglected in the set of differential equations governing the dynamics of CheA and CheY forms.

3.3 Phosphorylation of response regulator CheB

The differential equations for the phosphorylation of CheB are similar to those for CheY, however we assume that there is no re-binding to the P_2 domain after phosphorylation, i.e. $k_B^4 = 0$. In contrast to CheY, the phosphorylated CheB auto-dephosphorylates by a rate γ_B.

$$\frac{d}{dt}B = -k_B^1 B(A[P_2|P_1] + A[P_2|P_1p]) + k_B^2(A[P_2B|P_1] + A[P_2B|P_1p]) \\ - k_B B(A[P_2|P_1p] + A[P_2Y|P_1p] + A[P_2Yp|P_1p]) + \gamma_B Bp \quad (59)$$

$$\frac{d}{dt}Bp = k_B^5(A[P_2Bp|P_1p] + A[P_2Bp|P_1]) \\ + k_B B(A[P_2|P_1p] + A[P_2Y|P_1p] + A[P_2Yp|P_1p]) - \gamma_B Bp \quad (60)$$

$$\frac{d}{dt}A[P_2B|P_1] = k_B^1 BA[P_2|P_1] - (k_B^2 + k_A p_A)A[P_2B|P_1] + k_Y YA[P_2B|P_1p] \quad (61)$$

$$\frac{d}{dt}A[P_2B|P_1p] = k_B^1 BA[P_2|P_1p] + k_A p_A A[P_2B|P_1] - (k_B^2 + k_B^3 + k_Y Y)A[P_2B|P_1p] \quad (62)$$

$$\frac{d}{dt}A[P_2Bp|P_1] = k_B^3 A[P_2B|P_1p] - (k_A p_A + k_B^5)A[P_2Bp|P_1] + k_Y YA[P_2Bp|P_1p] \quad (63)$$

$$\frac{d}{dt}A[P_2Bp|P_1p] = k_A p_A A[P_2Bp|P_1] - (k_Y Y + k_B^5)A[P_2Bp|P_1p]. \quad (64)$$

We neglect terms $k_B BA[P_2B|P_1p]$ and $k_B BA[P_2Bp|P_1p]$, since $k_B B \ll k_Y Y$ and $A[P_2B|P_1p]+ A[P_2Bp|P_1p] \ll A[P_2|P_1p] + A[P_2Y|P_1p] + A[P_2Yp|P_1p]$.

3.4 Stationary state equations

We now want to derive equations which are valid in the stationary state. First, we introduce the state variables

$$A[P_2Y] = A[P_2Y|P_1] + A[P_2Y|P_1p] \quad (65)$$

$$A[P_2Yp] = A[P_2Yp|P_1] + A[P_2Yp|P_1p] \tag{66}$$

$$A[P_2] = A[P_2|P_1] + A[P_2|P_1p]. \tag{67}$$

Adding the corresponding Eqs. (55) + (56) and (57) + (58), respectively, gives us

$$\frac{d}{dt}A[P_2Y] = k_Y^1 A[P_2]Y - k_Y^3 A[P_2Y|P_1p] - k_Y^2 A[P_2Y] \tag{68}$$

$$\frac{d}{dt}A[P_2Yp] = k_Y^4 A[P_2]Yp + k_Y^3 A[P_2Y|P_1p] - k_Y^5 A[P_2Yp]. \tag{69}$$

In the stationary state, we get

$$A[P_2Y] = \frac{k_Y^1}{k_Y^2}A[P_2]Y - \frac{k_Y^3}{k_Y^2}A[P_2Y|P_1p] \tag{70}$$

$$A[P_2Yp] = \frac{k_Y^4}{k_Y^5}A[P_2]Yp + \frac{k_Y^3}{k_Y^5}A[P_2Y|P_1p]. \tag{71}$$

The conservation of all $A[P_2]$ domains and the Eqs. (70) and (71) give us

$$A[P_2] = A^T - A[P_2Y] - A[P_2Yp] \tag{72}$$

$$\approx \frac{A^T}{1 + Y/K_Y^D + Yp/K_{Yp}^D}, \tag{73}$$

where $K_Y^D = k_Y^2/k_Y^1, K_{Yp}^D = k_Y^5/k_Y^4$. The approximation becomes exact if $k_Y^2 = k_Y^5$ holds.

Using the definitions introduced above, we can rewrite Eq. (56) for $A[P_2Y|P_1p]$,

$$\frac{d}{dt}A[P_2Y|P_1p] = k_Y^1 A[P_2|P_1p]Y + k_A p_A (A[P_2Y] - A[P_2Y|P_1p]) - \left(k_Y^2 + k_Y^3 + k_Y Y\right) A[P_2Y|P_1p]. \tag{74}$$

Solving for $A[P_2Y|P_1p]$ in the stationary state and using Eq. (70), we get

$$A[P_2Y|P_1p] = \Omega_Y + \Gamma_Y A[P_2|P_1p] \tag{75}$$

$$\Omega_Y = \frac{\frac{k_Y^1}{k_Y^2} k_A p_A A[P_2]Y}{k_A p_A (1 + \frac{k_Y^3}{k_Y^2}) + k_Y^3 + k_Y Y + k_Y^2} \tag{76}$$

$$\Gamma_Y = \frac{k_Y^1 Y}{k_A p_A (1 + \frac{k_Y^3}{k_Y^2}) + k_Y^3 + k_Y Y + k_Y^2} \tag{77}$$

Similarly, we can formulate Eq. (58) for $A[P_2Yp|P_1p]$ as

$$\frac{d}{dt}A[P_2Yp|P_1p] = k_Y^4 A[P_2|P_1p]Yp + k_A p_A (A[P_2Yp] - A[P_2Yp|P_1p]) - (k_Y^5 + k_Y Y) A[P_2Yp|P_1p]. \tag{78}$$

This gives us, using Eqs. (71) and (75), $A[P_2Yp|P_1p]$ in the stationary state,

$$A[P_2Yp|P_1p] = \Omega_{Yp} + \Gamma_{Yp} A[P_2|P_1p] \tag{79}$$

$$\Omega_{Yp} = \frac{\frac{k_A p_A}{k_Y^5}\left(k_Y^3 \Omega_Y + k_Y^4 A[P_2]Yp\right)}{k_Y^5 + k_A p_A + k_Y Y} \tag{80}$$

$$\Gamma_{Yp} = \frac{\frac{k_A p_A}{k_Y^5} k_Y^3 \Gamma_Y + k_Y^4 Yp}{k_Y^5 + k_A p_A + k_Y Y}. \tag{81}$$

Finally, rewriting Eq. (54) for $A[P_2|P_1p]$,

$$\begin{aligned}\frac{d}{dt} A[P_2|P_1p] = k_A p_A (A[P_2] - A[P_2|P_1p]) - A[P_2|P_1p]((k_Y^1 + k_Y)Y + k_Y^4 Yp) \\ + k_Y^2 A[P_2Y|P_1p] + k_Y^5 A[P_2Yp|P_1p]\end{aligned} \tag{82}$$

and solving for $A[P_2|P_1p]$ in the stationary state, we get

$$A[P_2|P_1p] = \frac{k_A A[P_2] p_A + k_Y^2 A[P_2Y|P_1p] + k_Y^5 A[P_2Yp|P_1p]}{k_A p_A + k_Y^1 Y + k_Y Y + k_Y^4 Yp}. \tag{83}$$

Using Eqs. (75) and (79) yields

$$A[P_2|P_1p] = \frac{k_A p_A A[P_2] + k_Y^2 \Omega_Y + k_Y^5 \Omega_{Yp}}{k_A p_A + (k_Y^1 + k_Y)Y + k_Y^4 Yp - k_Y^2 \Gamma_Y - k_Y^5 \Gamma_{Yp}}. \tag{84}$$

Hence we can calculate $A[P_2|P_1p], A[P_2Y|P_1p]$ and $A[P_2Yp|P_1p]$ as a function of Y and Yp alone. The total amount of phosphorylated P1 domains is simply given by

$$A[P_1p] = A[P_2|P_1p] + A[P_2Y|P_1p] + A[P_2Yp|P_1p]. \tag{85}$$

Free CheY can be calculated from a conservation law

$$Y = Y^T - Yp - [YpZ] - A[P_2Y] - A[P_2Yp]. \tag{86}$$

We make the approximation $k_Y^2 \approx k_Y^5$ and use Eq. (73),

$$Y^T - Yp - [YpZ] - Y - \frac{A^T}{1 + Y/K_Y^D + Yp/K_{Yp}^D}\left(\frac{Y}{K_Y^D} + \frac{Yp}{K_{Yp}^D}\right) = 0. \tag{87}$$

This leads to an equation of second order in Y,

$$\begin{aligned} Y^2 + Y \overbrace{\left(A^T - Y^T + Yp + [YpZ] + K_Y^D\left(1 + \frac{Yp}{K_{Yp}^D}\right)\right)}^{b} \\ \underbrace{+ \frac{K_Y^D}{K_{Yp}^D}\left(A^T Yp + \left(K_{Yp}^D + Yp\right)\left(Yp + [YpZ] - Y^T\right)\right)}_{c} = 0. \end{aligned} \tag{88}$$

Only one of the solutions is physically meaningful

$$Y = \frac{1}{2}\left(-b + \sqrt{b^2 - 4c}\right). \tag{89}$$

The complex $[YpZ]$ is determined by solving Eq. (51) in the stationary state for $[YpZ]$ and using the conservation of phosphatases $Z^T = Z + [YpZ]$, yielding

$$[YpZ] = \frac{Z^T Yp}{K_Z^M + Yp}, \quad K_Z^M = \frac{k_Z^2 + k_Z^3}{k_Z^1}. \tag{90}$$

By adding Eqs. (50) + (51) we get an equation determining Yp in the stationary state

$$k_Y Y A[P_1p] + k_Y^5 A[P_2Yp] - k_Y^4 Yp A[P_2] - k_Z^3[YpZ] = 0. \tag{91}$$

Since we derived expressions giving us the functional dependence on Yp for all other variables, this is the only equation from our previous set of Eqs. (49) to (58) we have to solve numerically.

The equations for CheB are derived in a similar way. We introduce the state variables analogue to Eqs. (65) and (66)

$$A[P_2B] = A[P_2B|P_1] + A[P_2B|P_1p] \tag{92}$$
$$A[P_2Bp] = A[P_2Bp|P_1] + A[P_2Bp|P_1p], \tag{93}$$

and sum up the corresponding Eqs. (61) + (62) and (63) + (64), respectively:

$$\frac{d}{dt}A[P_2B] = k_B^1 BA[P_2] - k_B^2 A[P_2B] - k_B^3 A[P_2B|P_1p] \tag{94}$$
$$\frac{d}{dt}A[P_2Bp] = k_B^3 A[P_2B|P_1p] - k_B^5 A[P_2Bp]. \tag{95}$$

Similarly to Eq. (74) we can rewrite Eq. (62) for $A[P_2B|P_1p]$,

$$\frac{d}{dt}A[P_2B|P_1p] = k_B^1 BA[P_2|P_1p] + k_A p_A (A[P_2B] - A[P_2B|P_1p]) - (k_B^2 + k_B^3 + k_Y Y)A[P_2B|P_1p]. \tag{96}$$

Solving the stationary equations (94), (95) and (96), we get for $A[P_2Bp]$

$$A[P_2Bp] = B\Gamma_B \tag{97}$$
$$\Gamma_B = \frac{k_B^1 k_B^3 (k_B^2 A[P_2|P_1p] + k_A p_A A[P_2])}{k_B^5((k_B^2 + k_B^3)(k_B^2 + k_A p_A) + k_B^2 k_Y Y)}. \tag{98}$$

The equation governing free phosphorylated CheB, Eq. (60), can be rewritten in terms of $A[P_2Bp]$ and $A[P_1p]$

$$\frac{d}{dt}[Bp] = k_B^5 A[P_2Bp] + k_B BA[P_1p] - \gamma_B Bp. \tag{99}$$

Making the approximation $B \approx B^T - Bp$, we only have to solve (99) numerically for Bp in the stationary state.

3.5 Rate constants and concentrations

Rate constants are mostly taken from *in vitro* measurements (Dennis Bray's website: http://www.pdn.cam.ac.uk/groups/comp-cell/Rates.html) or if unknown set to reasonable values to reflect *in vivo* behavior as given by FRET measurements of kinase activity.

k_A	$\sim 40\,s^{-1}$	autophosphorylation of CheA
k_Y^1	$\sim 120\,\mu M^{-1}s^{-1}$	CheY to P_2 domain
K_Y^D	$\sim 1.3\,\mu M$	Dissociation constant of CheY at P_2 domain
k_Y^3	$\sim 800\,s^{-1}$	phosphotransfer of P_1p to CheY at P_2 domain
k_Y	$\sim 3.2\,\mu M^{-1}s^{-1}$	direct phosphotransfer from P_1p to CheY
k_Y^4	$\sim 120\,\mu M^{-1}s^{-1}$	CheYp to P_2 domain
K_{Yp}^D	$\sim 2.7\,\mu M$	Dissociation constant of CheYp at P_2 domain
k_Z^3	$\sim 8\,s^{-1}$	k_{cat} of CheYpCheZ
K_Z^M	$\sim 7.5\,\mu M$	Michaelis-Menten constant of CheYpCheZ
k_B^1	$\sim 0.4\,\mu M^{-1}s^{-1}$	CheB to P_2 domain
K_B^D	$\sim 1\,\mu M$	Dissociation constant of CheB at P_2 domain
k_B^3	$\sim 800\,s^{-1}$	phosphotransfer of P_1p to CheB at P_2 domain
k_B	$\sim 0.1\,\mu M^{-1}s^{-1}$	direct phosphotransfer from P_1p to CheB
k_B^4	$\sim 0\,\mu M^{-1}s^{-1}$	CheBp to P_2 domain
k_B^5	$\sim 4\,s^{-1}$	CheBp off P_2 domain
γ_B	$\sim 1\,s^{-1}$	autodephosphorylation of CheBp
k_R	$\sim 0.4\,s^{-1}$	CheR methylation rate of receptors
k_{Bp}	$\sim 17.9\,s^{-1}$	CheBp demethylation rate of receptors
p_A	$\sim 4 \cdot 10^{-2}$	fraction active receptors in the adapted state

Concentrations are calculated using protein copy numbers as given by Li and Hazelbauer [3] for rich media and assuming an effective cell Volume of $0.7\mu\text{m}^3$.

References

[1] Philippe Cluzel, Michael Surette, and Stanislas Leibler. An ultrasensitive bacterial motor revealed by monitoring signaling proteins in single cells. *Science*, 287:1652–1655, March 2000.

[2] Markus Kollmann, Linda Lovdok, Kilian Bartholome, Jens Timmer, and Victor Sourjik. Design principles of a bacterial signalling network. *Nature*, 438:504–507, November 2005.

[3] Mingshan Li and Gerald L. Hazelbauer. Cellular stoichiometry of the components of the chemotaxis signaling complex. *J. Bacteriol.*, 186:3687–3694, June 2004.
